# *Legionella pneumophila* CsrA regulates a metabolic switch from amino acid to glycerolipid metabolism

**DOI:** 10.1098/rsob.170149

**Published:** 2017-11-01

**Authors:** Ina Häuslein, Tobias Sahr, Pedro Escoll, Nadine Klausner, Wolfgang Eisenreich, Carmen Buchrieser

**Affiliations:** 1 Department of Chemistry, Biochemistry, Technische Universität München, Garching, Germany; 2 Institut Pasteur, Biologie des Bactéries Intracellulaires, Paris, France; 3 CNRS UMR 3525, Paris, France

**Keywords:** CsrA, *Legionella pneumophila*, isotopologue profiling, metabolism, nutrition, glycerolipid

## Abstract

*Legionella pneumophila* CsrA plays a crucial role in the life-stage-specific expression of virulence phenotypes and metabolic activity. However, its exact role is only partly known. To elucidate how CsrA impacts *L. pneumophila* metabolism we analysed the CsrA depended regulation of metabolic functions by comparative ^13^C-isotopologue profiling and oxygen consumption experiments of a *L. pneumophila* wild-type (wt) strain and its isogenic *csrA^−^* mutant. We show that a *csrA^−^* mutant has significantly lower respiration rates when serine, alanine, pyruvate, α-ketoglutarate or palmitate is the sole carbon source. By contrast, when grown in glucose or glycerol, no differences in respiration were detected. Isotopologue profiling uncovered that the transfer of label from [U-^13^C_3_]serine via pyruvate into the citrate cycle and gluconeogenesis was lower in the mutant as judged from the labelling patterns of protein-derived amino acids, cell-wall-derived diaminopimelate, sugars and amino sugars and 3-hydroxybutyrate derived from polyhydroxybutyrate (PHB). Similarly, the incorporation of [U-^13^C_6_]glucose via the glycolysis/Entner–Doudoroff (ED) pathway but not via the pentose phosphate pathway was repressed in the *csrA^−^* mutant. On the other hand, fluxes due to [U-^13^C_3_]glycerol utilization were increased in the *csrA*^−^ mutant. In addition, we showed that exogenous [1,2,3,4-^13^C_4_]palmitic acid is efficiently used for PHB synthesis via ^13^C_2_-acetyl-CoA. Taken together, CsrA induces serine catabolism via the tricarboxylic acid cycle and glucose degradation via the ED pathway, but represses glycerol metabolism, fatty acid degradation and PHB biosynthesis, in particular during exponential growth. Thus, CsrA has a determining role in substrate usage and carbon partitioning during the *L. pneumophila* life cycle and regulates a switch from amino acid usage in replicative phase to glycerolipid usage during transmissive growth.

## 1. Introduction

The Gram-negative, facultative intracellular pathogen *Legionella pneumophila* is widespread in natural and man-made aquatic systems, where it replicates within various free-living protozoan hosts like *Acanthamoeba castellanii* or *Hartmannella vermiformis* [1,2]. However, *L. pneumophila* is also able to infect human alveolar macrophages when contaminated aerosols are inhaled by a susceptible human host, causing Legionnaires' disease, a severe life-threatening pneumonia [3,4]. In both host systems, amoebae and macrophages, invasion occurs by phagocytosis followed by the establishment of an intracellular replication compartment, the *Legionella*-containing vacuole (LCV). The ability to replicate intracellularly is dependent on a functional Dot/Icm type IV secretion system (T4SS) that translocates over 300 effector proteins in the host cell, thereby triggering various processes such as the recruitment of vesicles derived from the endoplasmatic reticulum or the direct manipulation of many host cell signalling pathways [5–7].

Although several different morphological forms have been characterized [8], the *L. pneumophila* life cycle can mainly be described as biphasic. It consists of a (i) replicative form where bacteria are rod shaped, non-flagellated and are able to replicate in the LCV, and (ii) a transmissive form where the bacteria are flagellated and virulence and transmission factors are expressed allowing the infection of new host cells [9]. This biphasic life cycle is crucial for the fitness of the pathogen and is linked to its metabolism. Indeed, when nutrients are abundant, as in a host cell, the presence of amino acids triggers, for example, the differentiation of *L. pneumophila* to a replicative form [10,11]. Replication of *L. pneumophila* and thereby nutrient scavenging leads to amino acid consumption that triggers the switch to the transmissive form [12–14] that is expressing the virulence and transmission factors necessary to leave the spent host cell and search for a new one.

A link between the biphasic life cycle and the metabolism is also reflected in the life-stage-specific usage of carbon nutrients [15–17]. Although this pathogen is known to mainly use amino acids (e.g. serine) as carbon and energy source [18–20], the *L. pneumophila* genome analyses uncovered the presence of all enzymes of the glycolytic pathway and the Entner–Doudoroff (ED) pathway [21–23]. Indeed, their functionality in carbohydrate usage has been shown in transcriptome and proteome analyses, in *in vivo* experiments and by isotopologue profiling experiments, all confirming that *L. pneumophila* is able to metabolize glucose, whereby it predominantly uses the ED pathway [24,25]. In addition, *L. pneumophila* can use glycerol as a nutrient source. First hints came from early radio labelling experiments, *in vitro* studies as well as from transcriptome experiments that showed the upregulation of enzymes responsible for glycerol catabolism during intracellular growth in macrophages [20,26,27]. Recent *in vitro* isotopologue profiling studies of a *L. pneumophila* wt strain using [U-^13^C_3_]glycerol as a tracer demonstrated glycerol usage mainly in late growth phase where it serves as an additional substrate to feed the pentose phosphate pathway (PPP) and gluconeogenetic reactions [17]. Life-stage-specific substrate usage has also been demonstrated in labelling experiments with [U-^13^C_3_]serine and [U-^13^C_6_]glucose, highlighting that serine is more efficiently used in the replicative phase for energy generation via the tricarboxylic acid (TCA) cycle. Thus, the biphasic life cycle of *L. pneumophila* is represented by a switch from replicative to transmissive bacteria, a switch that is tightly linked to the metabolism and in particular to a life-cycle-specific substrate usage [15–17].

The metabolic changes occurring in the host cell during the intracellular replication of *L. pneumophila* are transmitted to regulatory systems and alternative sigma factors [28]*.* When nutrients are getting limited, the production of the stringent response messenger guanosine-3′,5′-bispyrophosphate (ppGpp) is induced, which in turn is activating among others the expression of RpoS and the two-component system LetA/LetS [10,29,30]. Consequently, the transcription of the three non-coding small RNAs RsmX, RsmY and RsmZ is induced, leading to the expression of transmissive traits and simultaneous repression of replicative traits due to binding to and thereby deactivating CsrA [31,32]. Thus CsrA is a key regulator of the switch from replicative to transmission competent bacteria [9,33–35]. CsrA prevents the translation of transmissive traits like motility, stress resistance or virulence during the exponential growth phase, while simultaneously activating replication [34–36]. The levels of *csrA* expression are furthermore regulated by the two-component system PmrA/PmrB that is essential for intracellular replication [31,37,38]. Another component of this regulatory network is the two-component system LqsTS/LqsR that is controlled by RpoS and LetA/LetS, and regulates quorum sensing in *L. pneumophila* [39,40].

Given the importance of CsrA in the life cycle switch between replicative and transmissive/virulent *L. pneumophila* and the fact that metabolic cues are regulating this biphasic life cycle, we investigated the role of the central regulator CsrA in the life-stage-specific carbon metabolism. *In vitro* ^13^C-isotopologue profiling and oxygen consumption experiments using the Seahorse Bioscience technology showed that the strong link between the *Legionella* life cycle and metabolism is reflected in different carbon source usage that is regulated by CsrA.

## 2. Results

### 2.1. Determination of *L. pneumophila* oxygen consumption reveals diminished energy generation by the *csrA* (*csrA*^−^) mutant strain

**Figure 1.**
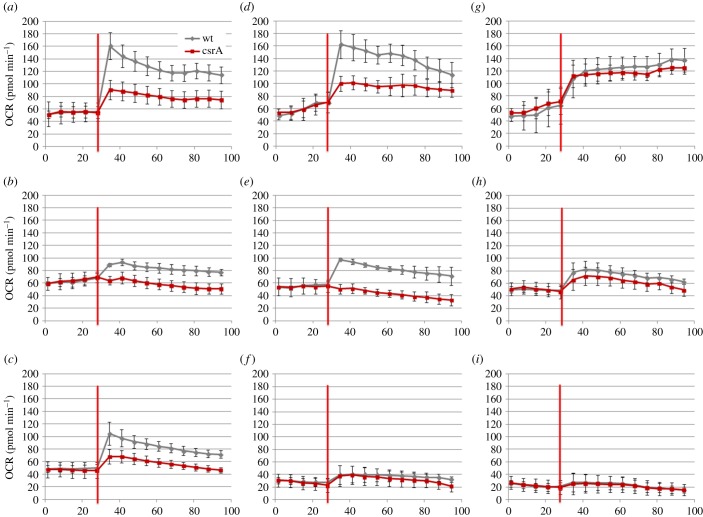
The *csrA^−^* mutant shows reduced oxygen consumption when compared with the wt strain. Changes in bacterial respiration (oxygen consumption rate, OCR) of *L. pneumophila* wt (grey) and *csrA* mutant (red) were measured as a function of various carbon sources. Bacteria remained untreated until the carbon source was added to the well (red line). The final concentration of the different substances added was as follows: (*a*) l-serine, 0.1 g l^−1^, (*b*) pyruvate, 0.2 g l^−1^, (*c*) α-ketoglutarate, 0.2 g l^−1^, (*d*) l-alanine, 0.1 g l^−1^, (*e*) palmitate, 0.025 g l^−1^, (*f*) d-glucose, 0.2 g l^−1^, (*g*) l-glutamate, 0.1 g l^−1^, (*h*) butanoate, 0.2 g l^−1^, (*i*) glycerol, 0.2 g l^−1^. Statistical analyses are shown in electronic supplementary material, table S13.

For a first evaluation of possible metabolic differences of the *L. pneumophila csrA*^−^ strain (truncated mutant after Tyr48 [35]), aerobic bacterial respiration was analysed by measuring the oxygen consumption rate (OCR) of the exponentially grown wild-type (wt) and the *csrA*^−^ strain in the presence of the substrates l-serine, l-alanine, l-glutamate, d-glucose, pyruvate, α-ketoglutarate, glycerol, butanoate or palmitate (figure 1). After poly-lysine coating of the *L. pneumophila* wt or *csrA*^−^ strain on micro-plates, bacterial respiration was measured using an extracellular flux analyser (Seahorse Bioscience). When l-serine was added as the substrate a clear increase in respiration was observed, confirming that serine is an important source for energy in *L. pneumophila* during exponential growth [17,24,41]. The OCR was significantly downregulated in the *csrA* mutant when compared with the wt, suggesting a positive effect of CsrA on the utilization of serine with regard to bacterial respiration (figure 1*a*). Similar results were obtained when pyruvate, α-ketoglutarate L-alanine or palmitic acid were added as substrate (figure 1*b*–*e*). By contrast, no difference between the OCR of the wt and the *csrA* mutant strain was observed when d-glucose, l-glutamate or glycerol were provided (figure 1*f*,*g*,*i*), suggesting no usage of these substrates in bacterial respiration. Interestingly, the OCR after injection of palmitate and to a lesser extent also of butanoate indicated usage of fatty acids (FAs) for aerobic respiration and revealed a positive effect of CsrA, in particular on the usage of palmitate (figure 1*e*,*h*). In contrast, unsaturated FAs like arachidonic acid or oleic acid showed no or a significantly reduced OCR of the wt strain (electronic supplementary material, figure S1). Metabolism and carbon flux from fatty acid degradation was not known in *L. pneumophila.* Our data suggest that *L. pneumophila* is able to metabolize saturated FAs such as palmitate and butanoate, but probably not unsaturated FAs like oleate or arachidonate. To study these findings in more detail we performed *in vitro* labelling experiments with [1,2,3,4-^13^C_4_]palmitate as described further below.

### 2.2. Serine uptake and metabolism are reduced in the *csrA* mutant strain

**Figure 2.**
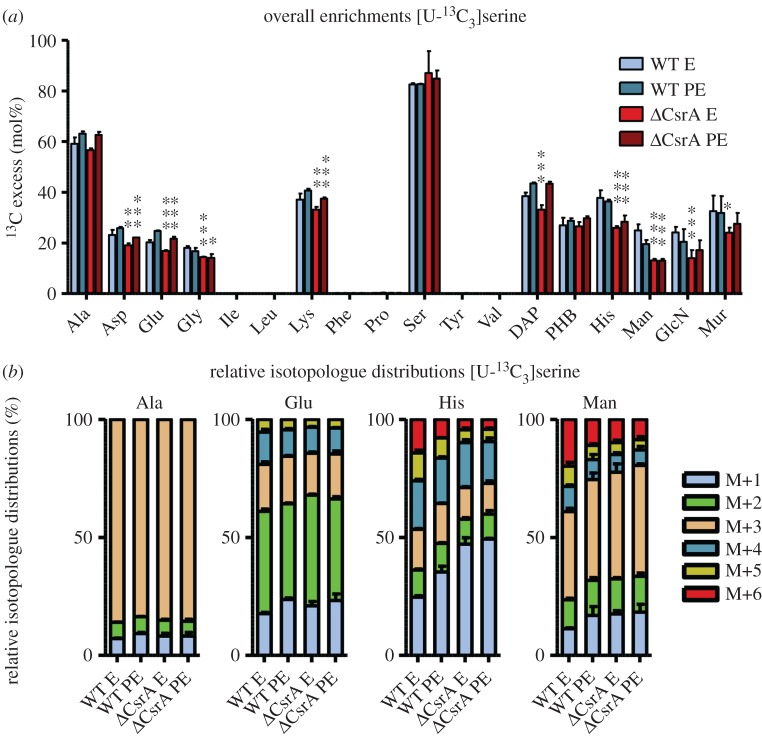
CsrA has regulatory impact on serine uptake and metabolism of *L. pneumophila*. (*a*) ^13^C excess (mol%) and (*b*) relative isotopologue distributions (%) in key metabolites from *L. pneumophila* wild-type and its *csrA* mutant grown in CE MDM supplemented with 6 mM [U-^13^C_3_]serine as tracer. Bacteria were harvested at the exponential (E) and post-exponential (PE) growth phase. ^13^C excess values (mol%) in protein-derived amino acids, diaminopimelic acid (DAP), polyhydroxybutyrate (PHB), mannose (Man), glucosamine (GlcN) and muramic acid (Mur) were determined by isotopologue profiling. Isotopologue distributions were determined for selected metabolites (Ala, Glu, His and Man). Shown are the relative fraction (in%) of isotopologues (M+1 to M+6). Error bars indicate standard deviations from six values (2 × biological replicates, 3 × technical GC/MS). Statistical significance is depicted as *p*-value (*p < 0.05, **p < 0.01 and ***p < 0.001). For numerical values, see electronic supplementary material, tables S4 and S8.

To investigate the role of CsrA in the carbon metabolism of serine, we performed labelling experiments with the wild-type and the *csrA* mutant grown in CE MDM medium supplemented with 6 mM [U-^13^C_3_]serine. Bacteria were harvested at E and PE growth phase and ^13^C overall enrichments and isotopologue distributions were determined in protein-derived amino acids that reflect in their precursors the core metabolic fluxes as detailed in table 1. In addition, products of key metabolic pathways and processes (table 1) were analysed, such as the carbon storage compound poly-3-hydroxybutyrate (PHB) and the cell wall components, diaminopimelic acid (DAP), mannose (Man), glucosamine (GlcN) and muramic acid (Mur), mainly found in peptidoglycans and lipopolysaccharides of the Gram-negative bacterial cell wall (figure 2; electronic supplementary material, figure S2). In the wt strain ^13^C enrichments were detected in the amino acids Ala, Asp, Glu, Gly, Lys, Ser and His as well as in PHB, DAP, Man, GlcN and Mur. High enrichment values were observed in Ala (besides Ser), but low ones in His or Man, which is in agreement with the previously proposed model of a bipartite metabolism of *L. pneumophila* wt, where Ser is mainly shuttled into the energy generating metabolic pathway of, for example, the TCA via pyruvate [17]. Overall enrichment values increased slightly from E to PE phase in the wt and the *csrA* mutant in Ala, Asp, Glu, Lys, DAP and PHB. In contrast, in Gly, His and sugars ^13^C enrichments remained constant or decreased from E to PE phase (electronic supplementary material, table S1).

**Table 1.** Detected metabolites and their central precursors used as markers for specific metabolic pathways. α-KG, α-ketoglutarate; OAA, oxaloacetic acid; PRPP, phosphoribosyl pyrophosphate; Man, mannose; GlcN, glucosamine; Mur, muramic acid; DAP, diaminopimelic acid; STE, stearic acid; LACT, lactate; EMP, Embden–Meyerhof–Parnas pathway; ED, Entner–Doudoroff pathway; PPP, pentose phosphate pathway.

detected marker metabolite	central precursor	metabolic pathway
Ala	pyruvate	EMP/ED
Ser	pyruvate	EMP/ED
Gly	Ser/pyruvate	glycine hydroxymethyltransferase
Val/Leu/Ile	pyruvate	pyruvate metabolism/TCA
Asp	OAA	TCA
Glu	α-KG	TCA
Pro	α-KG/Glu	TCA
DAP	pyruvate/Asp/OAA	TCA/cell wall biosynthesis
Lys	DAP/OAA	TCA/DAP biosynthesis
PHB	acetyl-CoA	energy and carbon storage
STE	acetyl-CoA	fatty acid biosynthesis
His	PRPP	PPP
Phe/Tyr	erythrose 4-phosphate/shikimate	shikimate pathway
Man	fructose/glucose	carbohydrate metabolism and interconversion
GlcN/Mur	Man/fructose	cell wall biosynthesis
LACT	pyruvate/methylglyoxal	pyruvate metabolism/methylglyoxal detoxification

In the *csrA* mutant (E phase) a slightly lower ^13^C incorporation (*p* < 0.01) was detected in Asp, Glu, Gly, Lys and DAP, and a strongly reduced enrichment (*p* < 0.001) was seen in His as well as in Man, GlcN and Mur when compared with the wt strain (electronic supplementary material, table S1). Minor, but similar effects were also detectable during the PE phase, indicating that serine uptake and metabolism are generally downregulated in the *csrA* mutant. However, in both growth phases, the carbon flux from ^13^C-serine into gluconeogenetic reactions and into the PPP was strongly affected by this mutation, as ^13^C enrichments were mainly reduced in key metabolites of these pathways. In contrast, ^13^C enrichments in metabolites related to the TCA cycle were only slightly changed, indicating smaller effects of CsrA on the carbon flux of serine into the TCA cycle (figure 2*a*).

The relative fractions (%) of isotopologues containing one, two, three, … , *n* ^13^C-atoms (M+1, M+2, M+3, … , M+*n*) in the ^13^C-enriched samples were then determined from the intensities of the respective MS signals in comparison to the respective unlabelled compound (figure 2*b* and electronic supplementary material, table S2). Ala and Glu showed similar ^13^C distributions in the wt and the *csrA*^−^ strain in both growth phases, but the isotopologue distribution in His and Man clearly differed in the mutant, especially during the E phase. The amount of M+6 and M+5 isotopologues was significantly reduced in the *csrA* mutant. The presence of high amounts of M+6 label in Man and high amounts of M+5 label in His is the result of a high carbon flux from serine into gluconeogenetic reactions and into the PPP, because a combination reaction of two fully labelled C_3_ compounds is required to afford these highly labelled isotopologues. The required fully labelled C_3_ precursors are synthesized via the formation of glyceraldehyde 3-phosphate (GAP), which is then shuffled into the PPP for His biosynthesis or used for the formation of sugars. Furthermore, high amounts of M+6 label in His also indicate a high carbon flux from serine into ATP biosynthesis, because one carbon atom in the biosynthesis of His is derived from one carbon of ATP. Thus, because the amount of M+5 and M+6 labelled isotopologue in His and Man was reduced in the *csrA* mutant, this suggests that CsrA negatively influences the carbon flux from serine through gluconeogenetic reactions into the PPP.

### 2.3. CsrA influences the carbon flux from glucose into the pentose phosphate pathway and Entner–Doudoroff pathways

Glucose as well as polysaccharides do not support the growth of *L. pneumophila* in culture [40,42]. Nevertheless, genes responsible for glucose metabolism (glycolysis and ED pathway) are present in the genome of this pathogen [21,22]. Indeed, recent labelling experiments revealed that *L. pneumophila* is able to effectively use glucose that is predominantly catabolized via the ED pathway and the PPP and only to a minor extent via glycolytic reactions, although the carbon flux from glucose into the TCA cycle for energy generation is low and it mainly serves biosynthetic processes [17,24,25]. However, the direct link between the ED pathway and the PPP is missing because the 6-phophogluconate dehydrogenase is not present in *L. pneumophila*.

**Figure 3.**
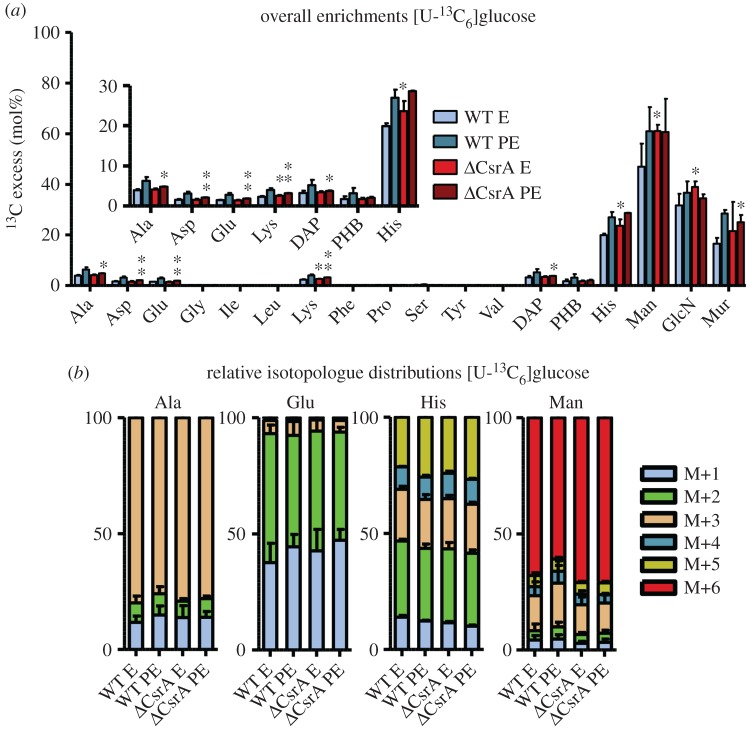
The carbon flux from glucose into the PPP and ED pathways as well as in the biosynthesis of sugars is higher in the *csrA* mutant. (*a*) ^13^C excess (mol%) and (*b*) relative isotopologue distributions (%) in key metabolites from *L. pneumophila* wild-type and its *csrA* mutant grown in CE MDM supplemented with 11 mM [U-^13^C_6_]glucose as tracers. Bacteria were harvested at the exponential (E) and post-exponential (PE) growth phase. ^13^C excess values (mol%) in protein-derived amino acids, diaminopimelic acid (DAP), polyhydroxybutyrate (PHB), mannose (Man), glucosamine (GlcN) and muramic acid (Mur) were determined by isotopologue profiling. Isotopologue distributions were determined for selected metabolites (Ala, Glu, His and Man). Shown are the relative fractions (in%) of isotopologues (M+1 to M+6). For a better illustration, metabolites with ^13^C excess values lower than 30% are shown in the figure inset. Error bars indicate standard deviations calculated from six values (two biological and three technical GC/MS replicates). Statistical significance is given as *p*-value (*p < 0.05, **p < 0.01 and ***p < 0.001). For numerical values see electronic supplementary material, tables S3 and S4.

To investigate whether CsrA might be involved in regulating this link we analysed the carbon metabolism of glucose in E and PE growth phase of *L. pneumophila* grown in CE MDM medium supplemented with 11 mM [U-^13^C_6_]glucose. High ^13^C enrichments were detected in His as well as in Man, GlcN and Mur. Lower enrichments were observed in Ala, Asp, Glu, Lys, DAP and PHB (figure 3; electronic supplementary material, figure S3). In most metabolites, the enrichment values increased from E to PE growth phase in the wt but less or not at all in the *csrA* mutant. Only ^13^C enrichments in Man and GlcN remained constant or slightly decreased in the *csrA* mutant from E to PE phase (electronic supplementary material, table S3).

The wt and the *csrA* mutant showed similar enrichment values during E phase for the TCA cycle related amino acids Ala, Asp, Glu, Lys as well as in DAP and PHB. Only His as well as Man and the cell wall sugar GlcN showed partly higher enrichments in the mutant (*p* < 0.05). This indicates that during E phase the carbon flux from glucose into the biosynthesis of these metabolites (via PPP and gluconeogenetic reactions) is slightly upregulated in the *csrA* mutant, whereas the carbon flux into the TCA cycle is hardly affected. During PE phase, ^13^C enrichment values decreased significantly in the *csrA* mutant in Ala, Asp, Glu, Lys, DAP and PHB (*p*-values <0.05). This was different in His, Man and GlcN where ^13^C incorporation was slightly higher or remained similar to the values of the wt (electronic supplementary material, table S3). Therefore, carbon flux from glucose into the TCA cycle seems to be predominantly affected by this mutation only during post-exponential growth. In contrast, gluconeogenetic reactions and carbon flux into the PPP seem to be already upregulated during E phase in the *csrA* mutant but not in the wt strain (figure 3*a*).

Similar results were obtained when analysing the isotopologue distributions, because the amounts of M+5 isotopologues in His and M+6 isotopologues in Man slightly increased in E and PE phase for the *csrA* mutant (figure 3*b*). The M+6 label in Man was derived from the direct conversion of fully labelled ^13^C-glucose into Man. In addition, M+6 isotopologues in Man can be formed via combination reactions of two fully labelled C_3_ precursors, which are previously built in glycolytic reactions or reactions of the ED pathway. M+5 label in His can be derived from fully labelled fructose 6-phosphate entering the PPP or again via combination reactions of two labelled precursors, which are recombined during gluconeogenetic reactions and/or in reactions of the PPP. Taken together, our results indicated, in contrast to what was observed for serine, a slightly higher carbon flux from glucose into the PPP and ED pathways as well as in the biosynthesis of sugars in the *csrA* mutant when compared with the wt strain (electronic supplementary material, table S4).

### 2.4. The glycerol metabolism is remarkably upregulated in the absence of CsrA

Early labelling experiments with [U-^14^C_3_]glycerol had suggested that glycerol is a potential nutrient for *L. pneumophila* [27]. Furthermore, transcriptome data showed that enzymes responsible for glycerol catabolism (*lpp1369*: glycerol kinase, *lpp2257*: glycerol 3-phosphate dehydrogenase) are upregulated during intracellular growth in macrophages [26]. Indeed, isotopologue profiling experiments of a wt strain demonstrated that *L. pneumophila* catabolizes this substrate, but exclusively at late stages of the developmental cycle, thereby mostly serving anabolic reactions in gluconeogenesis and the PPP [17].

**Figure 4.**
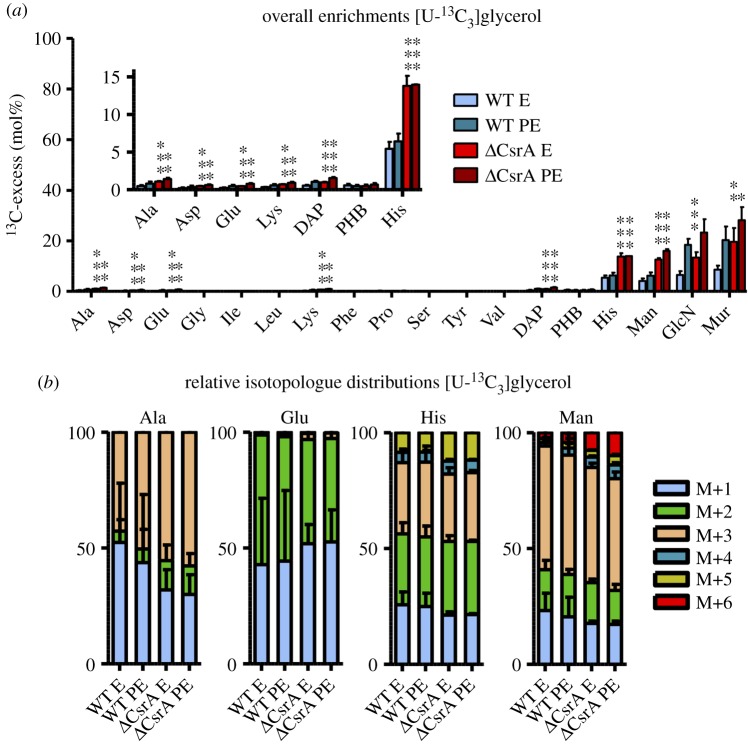
CsrA represses glycerol metabolism in E phase. (*a*) ^13^C excess (mol%) and (*b*) relative isotopologue distributions (%) in key metabolites from *L. pneumophila* wild-type and its *csrA* mutant grown in CE MDM supplemented with 50 mM [U-^13^C_3_]glycerol as tracer. Bacteria were harvested at the exponential (E) and post-exponential (PE) growth phase. ^13^C excess values (mol%) in protein-derived amino acids, diaminopimelic acid (DAP), polyhydroxybutyrate (PHB), mannose (Man), glucosamine (GlcN) and muramic acid (Mur) were determined by isotopologue profiling. Isotopologue distributions were determined for selected metabolites (Ala, Glu, His and Man). Shown are the relative fractions (in%) of isotopologues (M+1 to M+6). For a better illustration, metabolites with ^13^C excess values lower than 15% are shown in the figure inset. Error bars indicate standard deviations calculated from six values (two biological and three technical GC/MS replicates). Statistical significance is given as *p*-value (*p < 0.05, **p < 0.01 and ***p < 0.001). For numerical values see electronic supplementary material, tables S5 and S6.

To determine whether CsrA has a regulatory role on the metabolism of glycerol in *L. pneumophila*, we used labelling experiments with fully labelled ^13^C-glycerol. For comparisons, wild-type bacteria and a *csrA*^−^ strain were grown in CE MDM medium supplemented with 50 mM [U-^13^C_3_]glycerol, and cells were harvested at E and PE phase. Similar to the results obtained with labelled glucose, highest ^13^C enrichments were detected in His, which is a marker for the PPP (see also table 1) and in the sugars Man, GlcN and Mur. Low but significant ^13^C enrichments were also found in Ala, Asp, Glu, Lys, DAP and PHB, which are metabolites related to a carbon flux directed towards the TCA cycle (figure 4; electronic supplementary material, figure S4). In both labelling experiments (wt and *csrA* mutant), ^13^C overall excess values increased from the E to PE phase in all metabolites except for His in the *csrA*^−^ strain (electronic supplementary material, table S5).

Generally, ^13^C excess was higher in the *csrA*^−^ strain during both growth phases for almost every metabolite, when compared with the wt strain. The *csrA* mutant showed significant enrichment already during E phase in metabolites that are related to the TCA cycle. Specifically, ^13^C enrichment of His was increased by a factor of more than 2 in the mutant, rising from 5.45% (wt) to 13.79% (*csrA* mutant; *p* < 0.001) in the E phase and from 6.40% (wt) to 13.99% (*csrA* mutant; *p* < 0.001) in PE phase. Similar results were obtained for the cell wall sugars. This indicated that glycerol, which is only favoured in later growth phases in *L. pneumophila* wt, is already taken up and metabolized very efficiently during E phase in the *csrA* mutant. Furthermore, the glycerol metabolism and probably also incorporation of this substrate are dramatically upregulated in the *csrA*^−^ strain. This clearly demonstrates that CsrA has a negative, regulatory effect on the metabolism of glycerol in *L. pneumophila,* as the glycerol metabolism in general and predominantly the carbon flux into gluconeogenetic reactions and into the PPP seem to be affected (figure 4*a*).

This observation was also supported by the analyses of the isotopologue distributions in the respective metabolites (figure 4*b*; electronic supplementary material, table S6). For example, the amount of M+3 label in Ala increased in the *csrA* mutant. Since this isotopologue is directly derived from fully labelled glycerol, which is converted into pyruvate and subsequently used in Ala biosynthesis, this again suggested an upregulated incorporation and metabolism of glycerol in the absence of CsrA. Fully labelled pyruvate can then be used to build fully labelled acetyl-CoA, which is subsequently shuttled into the TCA cycle. Then, M+2 label is transferred to amino acids like Glu and Asp, which are directly derived from the TCA intermediates *α*-ketoglutarate and oxaloacetate. Therefore, the increased ^13^C overall enrichment in these metabolites is based on higher amounts of M+2-labelled acetyl-CoA, which is used for their biosynthesis via the TCA cycle in the *csrA*^−^ strain. The isotopologue distributions in metabolites related to gluconeogenesis (Man) and the PPP (His) differed also, because higher amounts of M+6 and M+5 label in E as well as in PE phase were present in the *csrA* mutant. As in experiments with fully labelled ^13^C-glucose, these multiple labelled isotopologues can only be formed via combination reactions of two fully labelled C_3_ precursors in gluconeogenetic reactions to yield M+6 label in Man. Likewise, M+5-labelled His can be derived from fully labelled fructose 6-phosphate entering the PPP or via combination reactions of two labelled precursors during reactions in the PPP. In summary, the glycerol metabolism is highly upregulated in the absence of CsrA, because the carbon flux from exogenous glycerol predominantly into metabolites derived from gluconeogenetic reactions and the PPP, increased in the mutant. This indicates that CsrA has an inhibitory effect on glycerol usage especially during the replicative phase.

### 2.5. Differential substrate usage analyses indicates that CsrA activates the tricarboxylic acid cycle and represses glycerol metabolism in E phase

**Figure 5.**
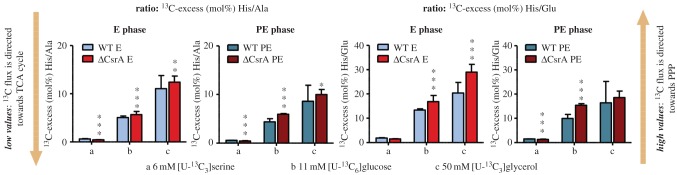
Differential substrate usage of *L. pneumophila* wild-type and its *csrA* mutant. Shown are ratios of ^13^C excess in His (PPP) to Ala (pyruvate) or Glu (TCA cycle), respectively. Ratios were calculated for experiments with *L. pneumophila* wt and its *csrA* mutant grown with (*a*) ^13^C-serine, (*b*) ^13^C-glucose and (*c*) ^13^C-glycerol for E phase and PE phase respectively. Mean values were calculated from matrix calculations (resulting in 36 data points) for possible His/Ala and His/Glu ratios with six datasets for His, Ala and Glu, respectively (two biological replicates; 2 × 3 technical replicates). The standard deviation was calculated from the resulting 36 His/Ala or His/Glu ratios, respectively. Statistical analysis was performed using two-tailed unpaired Student's *t*-test for the analysis of differences between the wild-type and the *csrA* mutant at the E and PE growth phase. Statistical significance is given as *p*-value (*p < 0.05, **p < 0.01 and ***p < 0.001). For numerical values see electronic supplementary material, tables S7 and S8.

To better visualize the metabolic changes that depend on CsrA, we calculated the ratios of ^13^C enrichments in marker metabolites between the wt and the *csrA* mutant strain. His was chosen as a marker for PPP, because PRPP derived from the PPP is a precursor for His. Ala and Glu were chosen as indicators for the TCA cycle, because Ala is derived from pyruvate and Glu from α-ketoglutarate, a direct intermediate of the TCA cycle (see also table 1). The two ratios ^13^C excess (mol%) His/Ala and ^13^C excess (mol%) His/Glu were calculated for the *L. pneumophila* wt and the *csrA*^−^ strain at the E and PE growth phase, respectively. High values are indicating an intense carbon flux directed towards gluconeogenetic reactions and into the PPP, whereas low values are representing a high carbon flux of the respective substrate into the TCA cycle for energy generation. Ratios were calculated for labelling experiments with [U-^13^C_3_]serine, [U-^13^C_6_]glucose and [U-^13^C_3_]glycerol (figure 5).

For the ^13^C-serine labelling experiments, these ratios were low in the wt and the mutant. Interestingly, the ratios were significantly reduced in the mutant, indicating that there is a higher carbon flux from serine into the TCA than into the PPP during both growth phases due to the *csrA* mutation (e.g. His/Ala: wt E: 0.64, *csrA^−^* E: 0.46; *p* < 0.001 and wt PE: 0.58, *csrA^−^* PE: 0.45; *p* < 0.001) (figure 5; electronic supplementary material, tables S7 and S8).

In contrast, experiments with ^13^C-glucose revealed high values for these ratios. This is in line with previous results obtained for a wt strain, which showed that glucose is predominantly shuttled into the PPP, the ED pathway and in glycolytic reactions [17]. In the *csrA*^−^ strain at both growth phases, these values were increased (e.g. His/Ala: wt E: 5.05, *csrA^−^* E: 5.70; *p* < 0.001 and wt PE: 4.36, *csrA^−^* PE: 5.95; *p* < 0.001), showing that CsrA has a regulatory impact on the carbon flux from glucose towards anabolic processes (figure 5).

Interestingly, the highest ratios were calculated for the experiments with [U-^13^C_3_]glycerol, which indicates that carbon flow from this nutrient is mainly directed to the PPP in both the wt and the *csrA* mutant. Importantly, these values increased in E and PE phase dependent on CsrA. The incorporation and metabolism of glycerol is strongly upregulated in the absence of CsrA while the carbon flux is mostly restricted to reactions in the PPP (figure 5). Thus, CsrA clearly represses glycerol uptake and/or metabolism towards PPP in *L. pneumophila* wt, especially during early growth phases where high levels of this regulator are present in the bacteria.

### 2.6. Fatty acids are preferentially used for poly-hydroxybutyrate biosynthesis dependent on the regulatory function of CsrA

The energy storage compound PHB that appears in granules predominantly in the transmissive growth phase is essential for long-term survival of *L. pneumophila* in the environment [42,43]. It is synthesized in numerous bacteria via (R)-3-hydroxybutanoyl-CoA, which is built from two acetyl-CoA molecules [16,44,45]. In a *L. pneumophila* wt strain it was shown that serine, but also glucose, is used to build acetyl-CoA*,* which is subsequently shuttled into the biosynthesis of PHB. Thereby, serine is partly used for PHB biosynthesis during replication, while glucose serves PHB biosynthesis at a later growth phase [16,17]. Here we analysed the putative role of CsrA as regulator of the time-dependent biosynthesis of this storage compound.

Analyses of the *L. pneumophila csrA*^−^ strain had revealed significantly higher concentrations of this storage compound during E and stationary growth phase and has identified numerous enzymes involved in the biosynthesis of PHB as direct targets of CsrA [35]. However, labelling experiments with [U-^13^C_3_]serine, [U-^13^C_6_]glucose and [U-^13^C_3_]glycerol did not show significantly increased ^13^C incorporation into PHB in the *csrA*^−^ strain (figure 2–4). Therefore, we hypothesized that another carbon source might serve for the biosynthesis of the higher amount of PHB in the *csrA* mutant.

**Figure 6.**
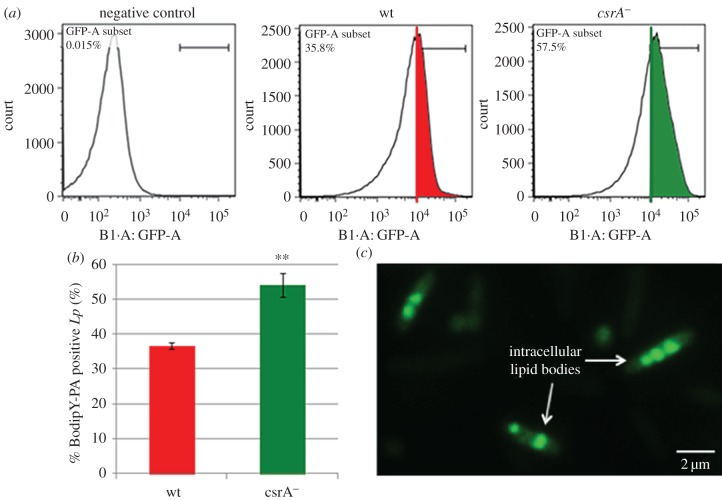
Significant increase of palmitate accumulation in the *csrA* mutant. Staining and quantification of palmitate incorporation was done by flow cytometry using Bodipy FL C16 and exponentially grown wt and *csrA*-mutant strains were compared. (*a*) To discriminate single-cell bacteria FSC A versus SSC-A and SSC-A versus SSC-H subsets were used to quantify fluorescence (530 ± 30 nm band pass filter). The threshold of PHB positive cells was determined by using unstained *L. pneumophila* cells as negative control. (*b*) In the absence of CsrA, a slight but significant shift to higher energies was observed equivalent to a higher percentage of Bodipy-palmitate positive bacteria. This indicates that palmitate uptake/accumulation might be favoured in the *csrA*^−^mutant compared with the wt. Each value represents the mean ± s.d. of three independent experiments. (*c*) Images from fluorescence microscopy suggest that *L. pneumophila* wt stores Bodipy-palmitate in intracellular lipid inclusion bodies, (scale bar, 2 µm).

Since all enzymes responsible for fatty acid degradation leading to acetyl-CoA, which can subsequently be used for the biosynthesis of PHB, are present in *L. pneumophila* [21–23], FAs could represent the carbon source for PHB biosynthesis. Indeed, when using fluorescently labelled palmitate (Bodipy FL C16) *in vivo,* we observed that *L. pneumophila* is able to take up and accumulate palmitate intracellularly. Furthermore, analyses by flow cytometry showed a slight but significant increase of palmitate accumulation in the *csrA* mutant when compared with the wt strain (figure 6*a*,*b*). This confirms our model that CsrA seems to have a negative regulatory effect on palmitate uptake and/or short-term utilization in oxidative phosphorylation (figure 1*e*). However, as judged by fluorescence microscopy, in both the wt and *csrA*^−^ strain, the FAs were stored in intracellular inclusion bodies similar to what is known for other bacteria such as *Mycobacterium tuberculosis* (figure 6*c*). Most interestingly, in *M. tuberculosis*, triacylglycerols released from the host cell are the main energy source, and the synthesis and accumulation of lipid droplets inside the bacteria is closely related to stress response, antibiotic resistance and dormancy [46].

**Figure 7.**
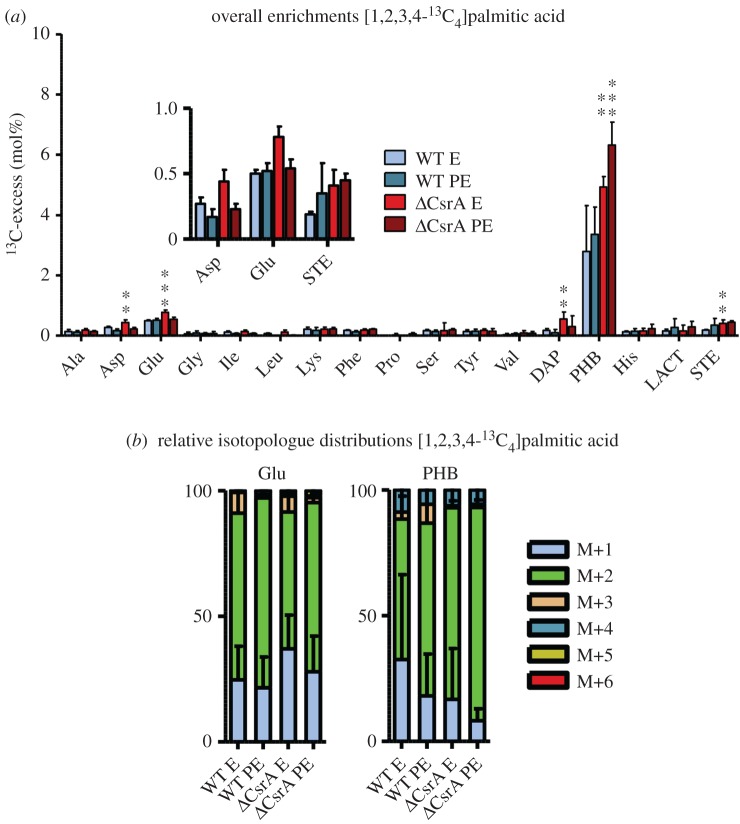
Palmitic acid predominantly serves as carbon source for the PHB biosynthesis in the wt. (*a*) ^13^C excess (mol%) and (*b*) relative isotopologue distributions (%) in key metabolites from *L. pneumophila* wild-type and its *csrA* mutant grown in CE MDM supplemented with 0.8 mM [1,2,3,4-^13^C_4_]palmitic acid as tracers. Bacteria were harvested at the exponential (E) and post-exponential (PE) growth phase. ^13^C excess values (mol%) in protein-derived amino acids, diaminopimelic acid (DAP), polyhydroxybutyrate (PHB), lactate (LACT) and stearic acid (STE) were determined by isotopologue profiling. Isotopologue distributions were determined for selected metabolites (Glu and PHB). Shown are the relative fractions (in%) of isotopologues (M+1 to M+6). For a better illustration, metabolites with ^13^C excess values of lower than 1% are shown in the figure inset. Error bars indicate standard deviations calculated from six values (two biological and three technical GC/MS replicates). Statistical significance is given as *p*-value (*p < 0.05, **p < 0.01 and ***p < 0.001). For numerical values, see electronic supplementary material, tables S9 and S10.

To further analyse the impact of long-chain FAs, we performed for the first time also labelling experiments with [1,2,3,4-^13^C_4_]palmitic acid as ^13^C-carbon tracer. The *L. pneumophila* wt and the *csrA* mutant were grown in CE MDM medium with [1,2,3,4-^13^C_4_]palmitic acid and were harvested at E and PE phase. ^13^C enrichments and isotopologue profiles were determined in protein-derived amino acids, DAP and PHB as well as in lactate and stearic acid (figure 7; electronic supplementary material, tables S9 and S10). High ^13^C enrichments were detected exclusively in the storage compound PHB. This demonstrates for the first time the effective usage of a fatty acid in *L. pneumophila* and that acetyl-CoA derived from fatty acid degradation indeed serves predominantly for the biosynthesis of the storage compound PHB. Minor, but significant enrichments were also detected in Glu in the wt strain, indicating that minor amounts of labelled acetyl-CoA are also shuffled into the TCA cycle. ^13^C enrichments in PHB significantly increased in the *csrA*^−^ strain in E phase (wt: 2.79%, *csrA* mutant: 4.93%; *p* < 0.05) and PE phase (wt: 3.36%, *csrA* mutant: 6.32%; *p* < 0.001) (electronic supplementary material, table S9). During E phase, also ^13^C carbon flux into the TCA cycle was upregulated in the *csrA*^−^ strain, because higher amounts of labelling were detected in Glu (wt: 0.50%, *csrA* mutant: 0.78%; *p* < 0.001) (electronic supplementary material, table S10). Additionally, a slightly increased carbon flux also occurred into the *de novo* biosynthesis of stearic acid in the *csrA* mutant during E phase (*p* < 0.01) (figure 7). In conclusion, CsrA is directly involved in the regulation of the growth phase-dependent biosynthesis of PHB by reducing carbon flux from FAs towards acetyl-CoA.

## 3. Discussion

The RNA-binding protein CsrA post-transcriptionally controls metabolism, motility and virulence in many bacteria [36,47]. *Legionella pneumophila* CsrA is known to play a crucial role in the regulation of the bacterium's biphasic life cycle, which is reflected in the expression of replicative traits during early stages of infection and virulence/transmissive traits during late stages of the infection [9]. However, among the over 500 CsrA targets of *L. pneumophila* identified recently, many have functions in the carbon, amino acid and fatty acid metabolism as well as energy transfer and the biosynthesis of cofactors, vitamins and secondary metabolites [35].

Here we unveil, by using isotopologue profiling of a wt and a *csrA* mutant strain, and the use of serine, glucose, glycerol or palmitic acids as ^13^C-carbon tracers, that CsrA has significant regulatory functions in many metabolic pathways of *L. pneumophila*. We show that: (i) CsrA activates serine uptake and metabolism; (ii) CsrA influences the carbon flux through the glycolysis/ED pathways and gluconeogenesis; (iii) CsrA activates the flux towards the TCA; and (iv) CsrA has a negative, regulatory effect on the metabolism of glycerol*.* Furthermore, by using for the first time [1,2,3,4-^13^C_4_]palmitic acid as a ^13^C-carbon tracer we revealed that carbon from palmitic acids are preferentially used for PHB biosynthesis dependent on the regulatory function of CsrA. Finally, the determination of the oxygen consumption rate of *L. pneumophila* wt compared with the *csrA* mutant when l-serine, l-alanine, pyruvate, α-ketoglutarate or palmitic acid were used as carbon source, disclosed reduced energy generation by the *csrA* mutant. This is in line with our recent results showing, for example, that genes coding for functions important for serine incorporation (*lpp2269*) or metabolism (*lpp0854*) are targets of CsrA [35].

**Figure 8.**
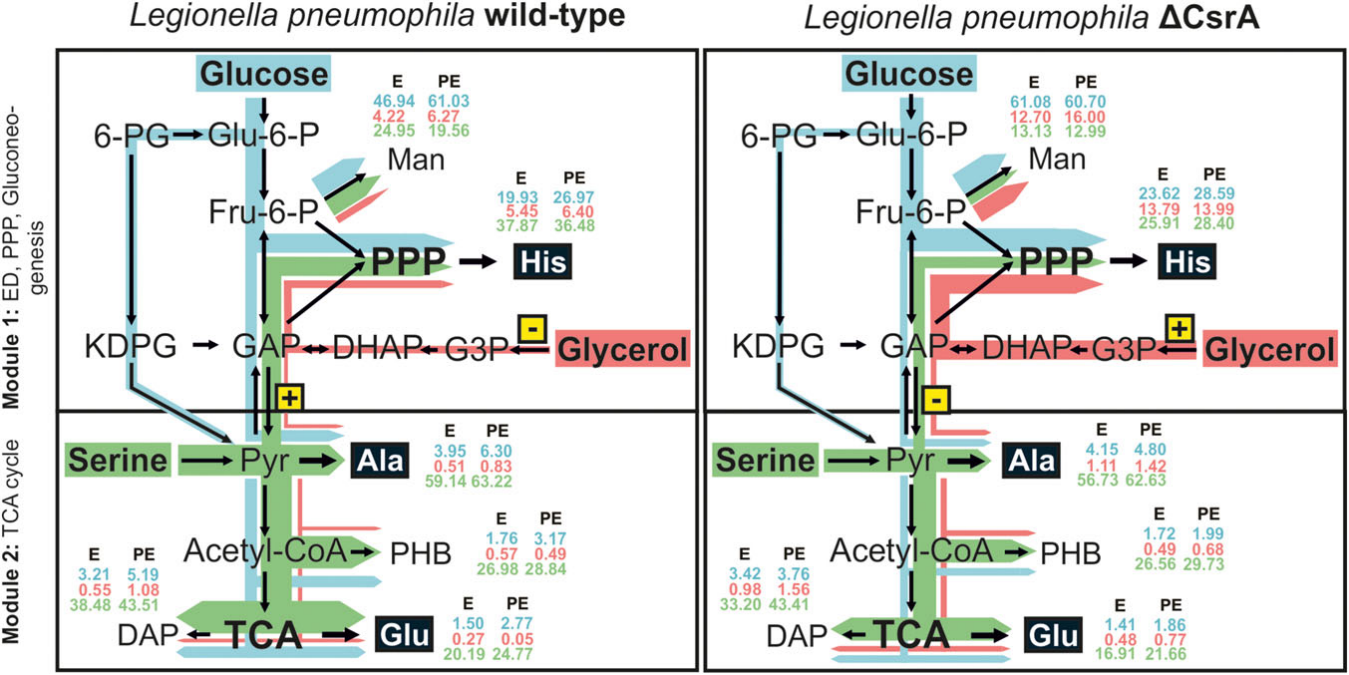
CsrA plays a major role in the regulation of the bipartite metabolism of *L. pneumophila*. Module 1 comprises the ED pathway, gluconeogenesis and the pentose phosphate pathway. Module 2 includes the TCA cycle. ^13^C enrichment values for E and PE phase from the three labelling experiments (blue: glucose; red: glycerol; green: serine) are shown for Man, His, Ala, Glu and DAP. Coloured arrows represent main carbon fluxes from the three different nutrients in *L. pneumophila* wt (left) compared with its *csrA*-strain (right). Thickness of arrows indicates relative carbon flow intensities from the three different substrates, respectively, into metabolic marker metabolites His (PRPP/PPP), Ala (pyruvate/TCA) and Glu (α-KG/TCA). Thereby, the thickness of an arrow is not comparable between the different substrates, but illustrates the main carbon fluxes and differences between the two strains (wild-type compared with *csrA*-strain). Serine is mainly used in the energy generating part of metabolism (module 2) in the wt and *csrA* mutant, though serine metabolism is reduced in the *csrA* mutant. Also the carbon flux from serine into the upper part of metabolism is lower in the *csrA*-strain. Metabolic flow of glucose is mainly restricted to module 1, though carbon flux also occurs into the energy generating part of metabolism in the wt. In the *csrA* mutant carbon flux from glucose into the PPP was not affected, while glucose metabolism in the TCA cycle was reduced. The *csrA* mutation drastically changed glycerol metabolism in *L. pneumophila*. In the wt, glycerol metabolism is very low and almost exclusively restricted to the upper part of metabolism (module 1). In the *L. pneumophila csrA*-strain, glycerol metabolism is dramatically upregulated. Carbon flow also occurs predominantly into module 1, but carbon flow from glycerol into the TCA cycle has also been detectable. Framed and highlighted in yellow plus or minus signs are representing enzymes, which are upregulated [+] or downregulated [−] dependent on CsrA levels. In the *L. pneumophila* wt strain enzymes of the second part of the glycolysis are induced by CsrA (*lpp0151:* pyruvate kinase*, lpp0152:* phosphoglycerate kinase*, lpp0153:* glyceraldehyde 3-phosphate dehydrogenase), whereas the glycerol kinase is negatively (*lpp1369*) affected. PRPP, phosphoribosyl pyrophosphate; α-KG, α-ketoglutarate; Glu-6-P, glucose-6-phosphate; Fru-6-P, fructose-6-phosphate; 6-PG, 6-phosphogluconate; KDPG, 2-keto-3-desoxy-phosphogluconate; GAP, glyceraldehyde-3-phosphate; DHAP, dihydroxyacetone phosphate; G3P, glycerol-3-phosphate; Pyr, pyruvate; PPP, pentose phosphate pathway; PHB, polyhydroxybutyrate; DAP, diaminopimelate; Man, mannose; ED pathway, Entner–Doudoroff pathway; TCA, tricarboxylic acid cycle.

Previous studies had revealed a bipartite metabolic network with life-stage-specific usages of amino acids, carbohydrates and glycerol as major substrates. For example, serine is predominantly used in the E growth phase for energy generation in the TCA cycle [17]. Here, we show that CsrA is an important regulator of the bipartite metabolism in *L. pneumophila.* The carbon flux into the energy generating part of the bipartite metabolism (module two) that is derived from glucose was reduced in the *csrA^−^* strain, whereas carbon flux into the upper part (module one) was not affected (figure 8).

This is in line with previous proteome and transcriptome data in which CsrA positively affects several enzymes of the carbon metabolism via direct interaction, including the ED pathway and the second part of the glycolysis, whereas a glucose transporter was negatively affected by CsrA [35] (electronic supplementary material, figures S5 and S6). As a result, even though the overall uptake of glucose might be induced in the absence of CsrA, the attenuation of the ED pathway and the second part of the glycolysis leads to a reduced carbon flux directed towards module 2. Thereby, the operon coding for glyceraldehyde 3-phosphate, phosphoglycerate kinase, pyruvate kinase (*lpp0151-lpp0154*), three enzymes of the glycolytic cascade and the transketolase, an enzyme of the PPP, seems to play a key role in the regulation of carbon flux partitioning between module one and module 2. Indeed, we have shown previously that this operon is directly targeted by CsrA, inducing the transcription of the three glycolytic enzymes, whereas regulation of the transcription level of the transketolase is not under the control of CsrA [35]. Thus, our isotopologue analysis is in line with this finding as in the absence of CsrA, the incorporation of glucose is induced and glyceraldehyde 3-phosphate built from glucose is shuttled without hindrance into the PPP, because reactions of the transketolase are not affected whereas pyruvate biosynthesis from glyceraldehyde 3-phosphate is reduced. Apparently, the regulation of this operon by CsrA does not only affect the formation of pyruvate via glyceraldehyde 3-phosphate, but also gluconeogenetic reactions in the opposite direction to form glyceraldehyde 3-phosphate from pyruvate, because *L. pneumophila* also uses gluconeogenesis [17]. Therefore, besides the reduced uptake and use of serine in the *csrA^−^* strain, the flux of ^13^C-serine into the upper part of the metabolism to generate histidine highlights the regulatory function of CsrA at the interface of the two modules of the bipartite metabolism.

Furthermore, experiments with ^13^C-glycerol revealed significant differences in the metabolism of the *csrA^−^* when compared with the wt strain. CsrA inhibits the glycerol metabolism until late growth phases where glycerol is then exclusively shuttled into module one for anabolic processes. Owing to this high incorporation and metabolic rate of glycerol in the mutant, increased ^13^C enrichment values were detectable in metabolites related to the TCA cycle, but the overall carbon flux was more restricted towards the upper part of the bipartite metabolism (module 1). This might be mainly due to the regulatory effect of CsrA on the operon *lpp0151-lpp0154* and additionally by its negative influence on the glycerol kinase (*lpp1369*) and glycerol-3-phosphate dehydrogenase (*lpp1368*), as shown in protein and transcript levels [35] (electronic supplementary material, figure S6).

Taken together, not only the developmental cycle is dependent on the CsrA regulatory system, but also the life-stage-specific metabolism of *L. pneumophila*. Thereby, CsrA is responsible for the upregulation of enzymes in the TCA cycle and the effective uptake and usage of serine. During later growth stages, this central regulator induces the metabolic flow from alternative carbon sources like glucose and more important glycerol, subsequently directing metabolism towards anabolic processes. Furthermore, CsrA seems to be a key player in the regulatory network of the bipartite metabolism, predominantly via the regulation of the operon *lpp0151-lpp0154,* which comprises enzymes at the interface of the two metabolic modules (ED, PPP, gluconeogenesis = module 1; TCA cycle = module 2) (figure 8).

**Figure 9.**
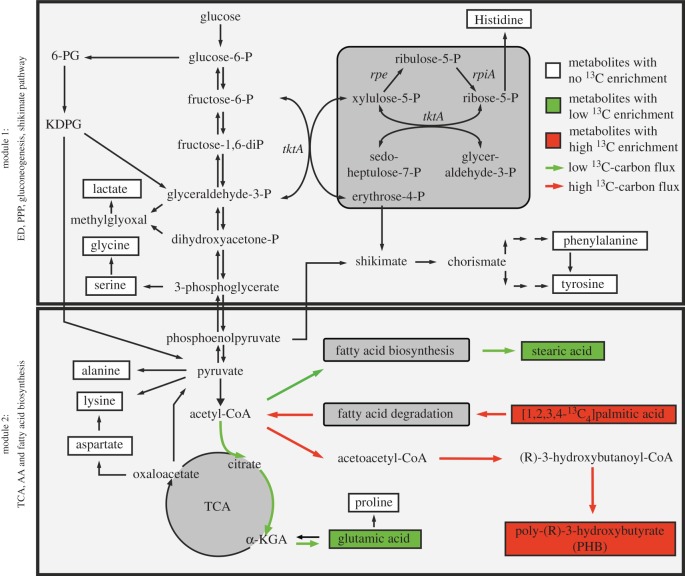
FAs are used nearly exclusively in later stages of the *L. pneumophila* life cycle. The metabolism of palmitic acid in *L. pneumophila* wt and its *csrA* mutant is different. Carbon flux from palmitic acid is exclusively restricted to module 2 in the *L. pneumophila* wt and the *csrA-*strain. Thereby, this precursor predominantly serves as carbon source for PHB biosynthesis (indicated in red). Only in the *csrA* mutant, a minor carbon flux into the TCA cycle and fatty acid biosynthesis was detectable (indicated in green).

Using labelling experiments with [1,2,3,4-^13^C_4_]palmitic acid as tracer, we show for the first time the effective usage of a long-chain fatty acid by *L. pneumophila* as nutrient. It primarily serves for the biosynthesis of the storage compound PHB, which is predominantly built in later growth phases [42,43] (figure 9). This metabolic link between β-oxidation and PHB biosynthesis has also been observed in other bacteria, such as *Pseudomonas putida* [48], and was hypothesized to be present in *L. pneumophila* [15]. The presence of many phospholipases in the genome of *L. pneumophila* might also hint at the utilization of FAs as nutrients. Some of them are virulence factors and are expressed at later growth phase further indicating a life stage specific regulation of fatty acid degradation [49]. Furthermore, short-chain FAs also trigger the developmental switch between the replicative and the transmissive growth phase of *L. pneumophila,* suggesting that fatty acid metabolism and virulence are directly connected [13]. Studies on cell-associated enzymes revealed lipolytic activities, whereby hydrolysis of eukaryotic membrane constituents could be an important virulence factor of *L. pneumophila* [50]. Indeed, the high phospholipase potential that is present in this pathogen is involved in the disease development [49]. The cell-associated haemolytic phospholipase A (PlaB), which preferably hydrolyses long-chain FAs (more than C_12_), encodes the major lipolytic activity in *L. pneumophila* [50]. Thus, this intracellular pathogen might lyse the membrane of its host during transmissive growth phase and subsequently use the released components (preferably palmitic acid and glycerol) for anabolic processes and also PHB biosynthesis. Nevertheless, no utilization of palmitic acid for bacterial respiration was observed in the *csrA*^−^ strain, suggesting a positive effect of CsrA on energy metabolism or uptake of this fatty acid already during replication. However, proteome and transcriptome analysis demonstrated that most enzymes involved in PHB biosynthesis were negatively affected by CsrA, including acetoacetyl-CoA reductase enzymes (*lpp0620*, *lpp0621* and *lpp2322*) as well as a polyhydroxyalkanoate synthase (*lpp2038*) [35,51] (electronic supplementary material, figure S7). Most of them are directly targeted by CsrA, repressing expression of these enzymes during E phase in *L. pneumophila* wt. Interestingly, a long-chain fatty acid transport protein (*lpp1773*) is also targeted by this central regulator [35]. Indeed the role of CsrA in the coordination of fatty acid uptake, degradation and usage for PHB biosynthesis is also confirmed by our isotopologue profiling experiments, because higher enrichment values have been found in PHB in the labelling experiments with the *csrA* mutant. This clearly shows the inhibitory effect of this post transcriptional regulator on fatty acid degradation and simultaneously on PHB biosynthesis during exponential growth.

In summary, palmitic acid predominantly serves as carbon source for PHB biosynthesis in the wt, probably during the later growth phase when amino acids are depleted and the degradation of host glycerolipid may emerge as a main source for carbon in *Legionella*. As a consequence of this metabolic switch, the carbon of glycerol mainly enters the PPP and (cell wall) sugar synthesis, whereas the palmitic acid is used to fill up the carbon and energy storage supplies. Both are crucial to survive in the extracellular environment or might even act as a signal for *L. pneumophila* to prepare the evasion of its host. CsrA seems to act as an organizer for the switch between the usages of the different carbon source during the life cycle. During replication, CsrA supports that serine and probably other amino acids are used as energy and carbon source for the TCA cycle (module 2), concomitantly repressing glycerol usage and the production of PHB. In later growth phases, this inhibitory effect is reduced due to binding of CsrA to the sRNAs RsmX,Y,Z, leading to an increased metabolization of glycerol, which is together with glucose now the main source for the PPP (module 1), and an enhanced carbon flux via fatty acid degradation into PHB biosynthesis. Thus CsrA not only regulates the switch from replicative to transmission competent bacteria, but it governs in parallel the regulation of the bipartite metabolism of *L. pneumophila* and a metabolic switch from amino acid to carbohydrate/glycerolipid metabolism.

## 4. Material and methods

### 4.1. Bacteria, cells and growth conditions

In this study, the *L. pneumophila* Paris wt strain and its isogenic *csrA^−^* mutant strain [35] were grown in ACES-buffered yeast extract broth or an ACES-buffered charcoal-yeast extract agar under aerobic conditions at 37°C. For labelling experiments, bacteria were grown at 37°C in a carbon enriched minimal defined media (CE MDM-all components, except Fe-pyrophosphate are dissolved in 950 ml ddH_2_O. pH was adjust to 6.5 using KOH. Then Fe-pyrophosphate was dissolved and filled up to 1 l) (electronic supplementary material, table S11), as reported previously [17]. The *csrA*^−^ strain of *L. pneumophila* Paris was constructed by inserting an apramycin-resistance cassette after the amino acid Tyr48 of the *lpp0845* gene encoding the major CsrA in *L. pneumophila* Paris [35].

### 4.2. Oxygen consumption experiments

*Legionella pneumophila* Paris wt and *csrA*^−^ mutant were grown to exponential phase (OD_600_ = 2–2.5) in BYE, at 37°C and 170 rpm in a light-protected and humidity-controlled incubator shaker. After centrifugation, bacteria were resuspended in PBS (pH 7.4) to a final concentration of OD_600_ = 0.1 and 90 µl of the diluted cells were transferred to wells of the poly-d-lysine (PDL)-coated Microplate. To coat XF Cell Culture Microplate (Seahorse Bioscience), 15 µl of 1 mg ml^−1^ PDL in 100 mM Tris–HCl (pH 8.4) was added to each well, followed by overnight drying, and two dH_2_O rinses. PDL treatment has been shown to not significantly alter respiration in *Escherichia coli* [52]*.* Cells were attached by 10 min centrifugation at 4000 rpm in a benchtop swinging bucket centrifuge and afterwards the volume in each well was raised to 175 µl PBS. Bacterial respiration, expressed as oxygen consumption rates (OCR), was quantified using an XFe96 Extracellular Flux Analyzer (Seahorse Bioscience) according to the manufacturer's instructions. Basal OCR was measured for approximately 30 min to acquire a baseline prior to the injection of compounds. The different substrates were solubilized in PBS and 25 µl were injected to the reaction chamber at final concentrations of l-serine, l-alanine and l-glutamate: 0.1 g l^−1^; D-glucose, glycerol, butanoate, α-ketoglutarate and pyruvate: 0.2 g l^−1^; palmitate, oleate and arachidonic acid: 0.1 mM.

### 4.3. Isotopologue labelling experiments of *L. pneumophila*

^13^C-labelled precursors used in this study ([U-^13^C_3_]serine, [U-^13^C_6_]glucose, [U-^13^C_3_]glycerol and [1,2,3,4-^13^C_4_]palmitic acid) were ordered from Sigma-Aldrich. For labelling experiments, *L. pneumophila* wt and *csrA*^−^ strains were grown in CE MDM medium, whereas the amount of unlabelled serine, glucose and glycerol was displaced with 6 mM [U-^13^C_3_]serine, 11 mM [U-^13^C_6_]glucose and 50 mM [U-^13^C_3_]glycerol, respectively. For labelling experiments with [1,2,3,4-^13^C_4_]palmitic acid, bacteria were grown in CE MDM supplemented with additional 0.02% of labelled palmitic acid (0.8 mM). For these experiments, the respective *L. pneumophila* strain was grown over night in 50 ml unlabelled CE MDM medium. Afterwards, the inoculum was suspended in 50 ml of CE MDM comprising the respective ^13^C-tracer and diluted to an OD_600_ of 0.1. For every labelling experiment, bacteria were harvested at E and PE growth phase by centrifugation at 5000*g* for 5 min at 4°C. Cells were autoclaved for 30 min at 120°C, freeze-dried and stored at –80°C until further analysis.

### 4.4. Preparation of protein-derived amino acids, diaminopimelic acid and 3-hydroxybutyrate (derived from poly-hydroxybutyrate) for GC/MS analysis

For isotopologue profiling of protein-derived amino acid, DAP and PHB, 1 mg of the freeze-dried cell pellet was resolved in 0.5 ml of 6 N HCl. Sample was subsequently incubated at 105°C for 24 h, as described earlier [24]. Next, the HCl was removed under a stream of nitrogen at 70°C and the remaining residue was subsequently resolved in 200 µl acetic acid. Sample was purified using a cation exchange column of Dowex 50Wx8 (H^+^ form, 200–400 mesh, 5 × 10 mm), which was previously washed with 1 ml of MeOH and 1 ml ddH_2_O. Column was evolved with 2 ml of ddH_2_O (eluate 1) and 1 ml of 4 M ammonium hydroxide (eluate 2). Both samples were dried under a steam of nitrogen at 70°C. The remaining residue of eluate 1 was used for further analysis of PHB, whereas the residue of eluate 2 comprises protein-derived amino acids and DAP.

For derivatization of 3-hydroxybutyrate, derived from hydrolysis of PHB with HCl, dried samples of eluate 1 were dissolved in 100 µl of *N*-methyl-*N*-(trimethylsilyl)-trifluoroacetamide (Sigma). Afterwards, samples were incubated at 60°C in a shaking incubator at 110 rpm overnight. The resulting TMS derivative (TMS) of 3-hydroxybutyrate was used in following GC/MS analysis.

For analysis of protein-derived amino acids and DAP, the remaining residue of eluate 2 was resolved in a mixture of 50 µl dry acetonitrile and 50 µl *N*-(tert-butyldimethylsilyl)-*N*-methyl-trifluoroacetamide (Sigma) and incubated at 70°C for 30 min. The resulting TBDMS derivatives were used in following GC/MS analysis. Owing to degradation by acid hydrolysis, the amino acids tryptophan, arginine, methionine and cysteine could not be analysed. Furthermore, acid hydrolysation led to the conversion of glutamine and asparagine to glutamate and aspartate. Therefore, results for aspartate and glutamate correspond to asparagine/aspartate and glutamine/glutamate, respectively.

### 4.5. Preparation of lactate and stearate for GC/MS analysis

For isotopologue profiling of LACT and STE, 5 mg of the freeze-dried cell pellet was resolved in 1 ml of could MeOH. Then 800 mg of glass beads (0.25–0.05 mm) were added and mechanical cell lysis and extraction occurred using a ribolyser system (Hybaid) for 3 × 20 s at 6.5 m s^−1^. Afterwards, samples were centrifuged at 2300*g* for 10 min and the supernatant was dried under a stream of nitrogen. For analysis of LACT and STE, the remaining residue of the supernatant was resolved in a mixture of 50 µl dry acetonitrile and 50 µl *N*-(tert-butyldimethylsilyl)-*N*-methyl-trifluoroacetamide (Sigma) and incubated at 70°C for 1 h. The resulting TBDMS derivatives were used for GC/MS analysis.

### 4.6. Preparation of mannose for GC/MS analysis

For isotopologue profiling of mannose, 5 mg of the freeze-dried sample was resolved in 0.5 ml methanolic HCl (3 N) and incubated overnight at 80°C. After cooling down the sample, the supernatant was dried under a stream of nitrogen at 25°C. Next, 1 ml acetone containing 20 µl concentrated H_2_SO_4_ was added and derivatization occurred at 25°C for 1 h. Then 2 ml of saturated NaCl and 2 ml saturated Na_2_CO_3_ solution was added and extraction of this aqueous solution was done 2 × with 3 ml ethyl acetate. The combined organic phase was dried under a stream of nitrogen. In a second derivatization step, the dry residue was incubated overnight at 60°C with 200 µl of a 1 : 1 mixture of dry ethyl acetate and acetic anhydride. For GC/MS analysis, the derivatization reagents were removed and the remaining residue was resolved in 100 µl anhydrous ethyl acetate. Resulting diisopropylidene/acetate derivatives were used for GC/MS analysis.

### 4.7. Preparation and derivatization of the cell wall sugars glucosamine (GlcN) and muramic acid (Mur) for GC/MS analysis

For isotopologue profiling of GlcN and Mur, 15 mg of the freeze-dried sample was resolved in 0.5 ml of 6 N HCl and incubated overnight at 105°C. Following, solid components were removed by filtration and the filtrate was dried under a stream of nitrogen. One hundred microlitres of hexamethyldisilazane (HMDS) was added to the remaining residue and derivatization occurred for 3 h at 120°C. Resulting TMS derivatives were used for further GC/MS analysis.

### 4.8. Gas chromatography/mass spectrometry: analysis and isotopologue profiling

GC/MS analysis was performed with a QP2010 Plus gas chromatograph/mass spectrometer (Shimadzu) equipped with a fused silica capillary column (Equity TM-5; 30 m × 0.25 mm, 0.25 µm film thickness; SUPELCO) and a quadrupole detector working with electron impact ionization at 70 eV. An aliquot (0.1 to 6 µl) of the derivatized samples were injected in 1 : 5 split mode at an interface temperature of 260°C and a helium inlet pressure of 70 kPa. Selected ion monitoring was used with a sampling rate of 0.5 s and LabSolution software (Shimadzu) was used for data collection and analysis. All samples were measured three times for technical replicates. ^13^C excess values and isotopologue compositions were calculated as previously described [53]. This comprises (i) the detection of GC/MS spectra of unlabelled derivatized metabolites, (ii) determination of the absolute mass of isotopologue enrichments and distributions of labelled metabolites of the experiment, and (iii) correction of the absolute ^13^C incorporation by subtracting the heavy isotopologue contributions due to the natural abundances in the derivatized metabolites to calculate the isotopologue enrichments and distributions.

For amino acid and diaminopimelate analysis the column was first kept at 150°C for 3 min after injection of the sample. Then the column was developed with a temperature gradient of 7°C min^−1^ to a final temperature of 280°C, and this temperature was held for further 3 min. The amino acids alanine (6.7 min), glycine (7.0 min), valine (8.5 min), leucine (9.1 min), isoleucine (9.5 min), proline (10.1 min), serine (13.2 min), phenylalanine (14.5 min), aspartate (15.4 min), glutamate (16.8 min), lysine (18.1 min), histidine (20.4 min), tyrosine (21.0 min) and the cell wall component diaminopimelate (23.4 min) were detected and isotopologue calculations were performed with *m/z* [M-57]^+^ or *m/z* [M-85]^+^.

For analysis of LACT and STE, the column was kept at 100°C for 2 min after sample injection. Following, the column was developed with a first gradient of 3°C min^−1^ until a final temperature of 234°C. Subsequently, a second temperature gradient of 1°C min^−1^ until a final temperature of 237°C, and a third temperature gradient of 3°C min^−1^ to a final temperature of 260°C was performed. TBDMS derivatives of LACT (17.8 min) and STE (49.4 min) were detected. Isotopologue calculations were performed with *m/z* [M-57]^+^.

For diisopropylidene/acetate derivatives of the mannose, the column was kept at 150°C for 3 min after sample injection. Then the column was developed with a first temperature gradient of 10°C min^−1^ to a final temperature of 220°C, and a second temperature gradient of 50°C min^−1^ to a final temperature of 280°C. The final temperature was held for a further 3 min. Isotopologue calculations were performed with a fragment, which still contains all six C-atoms of mannose (*m/z* [M-15]^+^).

For analysis of the cell wall components GlcN and Mur the column was first kept at 70°C for 5 min. Following the column was developed with a temperature gradient of 5°C min^−1^ to a final temperature of 310°C. The final temperature was kept for an additional minute. Isotopologue calculations of the respective TMS derivatives were performed with *m/z* [M-452]^+^ and *m/z* [M-434]^+^. Retention times and mass fragments of derivatized metabolites that were used for all isotopologue calculations are documented in electronic supplementary material, table S12.

### 4.9. Fluorescence-labelled palmitate quantification

Bodipy FL C16 (Thermo Fisher Scientific) was solubilized in DMSO at a concentration of 1 mM. Bacterial cultures of wt and *csrA* mutant were grown in BYE until reaching exponential phase (OD 2). Then 500 µl were centrifuged for 3 min at 5000*g*. Pellets were washed once in 1 ml PBS, resuspended in PBS and adjusted to OD 1. Bacterial cells were incubated with 5 µM of Bodipy FL C16 for 10 min at 37°C. The cells were pelleted and washed three times with 1 ml PBS before resuspended in 400 µl PBS. Fluorescence was analysed with a MACSQuant flow cytometer (Miltenyi Biotec). Images of fluorescent Bodipy FL C16-labelled bacteria were acquired using an EVOS FL Cell Imaging System (Thermo Fisher) using the GFP LED cube (ex.470/22, em.510/42) at 100× magnification.

## Supplementary Material

Data tables Statistical analyses, and figures for the different isotopologue results

## Data accessibility

This article has no additional data.
