# Supplementary material for: Legionella pneumophila CsrA regulates a metabolic switch from amino acid to glycerolipid metabolism
Source: Open Biol. 2017 Nov 1;7(11):170149. doi: 10.1098/rsob.170149 (PMC5717341; doi:10.1098/rsob.170149)
Supplement: Data tables Statistical analyses, and figures for the different isotopologue results [file rsob170149supp1.docx]

**Supplemental material**

***Legionella pneumophila* CsrA regulates a metabolic switch from amino acid to glycerolipid metabolism**

Ina Häuslein^1^, Tobias Sahr^2,3^, Pedro Escoll^2,3^, Nadine Klausner^1^, Wolfgang Eisenreich^1*^, and Carmen Buchrieser^2,3*^

^1^Department of Chemistry, Biochemistry, Technische Universität München, Garching, Germany

^2^Institut Pasteur, Biologie des Bactéries Intracellulaires, Paris, France

^3^CNRS UMR 3525, Paris, France

**FIGURE S1.** Oxygen consumption experiments

**FIGURE S2.** ^13^C Isotopologue distributions from experiments with ^13^C-serine as precursor

**FIGURE S3.** ^13^C Isotopologue distributions from experiments with ^13^C-glucose as precursor

**FIGURE S4.** ^13^C Isotopologue distributions from experiments with ^13^C-glycerol as precursor

**FIGURE S5.** Regulation of serine metabolism by CsrA in *L. pneumophila*

**FIGURE S6.** Regulation of glucose and glycerol metabolism by CsrA in *L. pneumophila*

**FIGURE S7.** Regulation of PHB metabolism by CsrA in *L. pneumophila*

**Table S1**: ^13^C-Excess (mol%) of protein-derived amino acids, diaminopimelic acid (DAP), poly-hydroxybutyrate (PHB), mannose (Man), glucosamine (GlcN) and muramic acid (Mur) from experiments with *L. pneumophila* wild-type and its *csrA* mutant grown in CE MDM supplemented with 6 mM [U-^13^C_3_]serine.

**Table S2:** Relative fractions of isotopologues (mol%) of protein-derived amino acids, diaminopimelic acid (DAP), poly-hydroxybutyrate (PHB), mannose (Man), glucosamine (GlcN) and muramic acid (Mur) from experiments with *L. pneumophila* wild-type and its *csrA* mutant grown in CE MDM supplemented with 6 mM [U-^13^C_3_]serine.

**Table S3**: ^13^C-Excess (mol%) of protein-derived amino acids, diaminopimelic acid (DAP), poly-hydroxybutyrate (PHB), mannose (Man), glucosamine (GlcN) and muramic acid (Mur) from experiments with *L. pneumophila* wild-type and its *csrA* mutant grown in CE MDM supplemented with 11 mM [U-^13^C_6_]glucose**.**

**Table S4:** Relative fractions of isotopologues (mol%) of protein-derived amino acids, diaminopimelic acid (DAP), poly-hydroxybutyrate (PHB), mannose (Man), glucosamine (GlcN) and muramic acid (Mur) from experiments with *L. pneumophila* wild-type and its *csrA* mutant grown in CE MDM supplemented with 11 mM [U-^13^C_6_]glucose.

**Table S5**: ^13^C-Excess (mol%) of protein-derived amino acids, diaminopimelic acid (DAP), poly-hydroxybutyrate (PHB), mannose (Man), glucosamine (GlcN) and muramic acid (Mur) from experiments with *L. pneumophila* wild-type and its *csrA* mutant grown in CE MDM supplemented with 50 mM [U-^13^C_3_]glycerol.

**Table S6:** Relative fractions of isotopologues (mol%) of protein-derived amino acids, diaminopimelic acid (DAP), poly-hydroxybutyrate (PHB), mannose (Man), glucosamine (GlcN) and muramic acid (Mur) from experiments with *L. pneumophila* wild-type and its *csrA* mutant grown in CE MDM supplemented with 50 mM [U-^13^C_3_]glycerol.

**Table S7:** Ratio of ^13^C-excess in histidine to alanine calculated for E phase and PE phase phase for experiments with *L. pneumophila* wild-type and its *csrA* mutant grown in CE MDM with either 6 mM [U-^13^C_3_]serine, 11 mM [U-^13^C_6_]glucose or 50 mM [U-^13^C_3_]glycerol as tracers.

**Table S8:** Ratio of ^13^C-excess in histidine to glutamine calculated for E phase and PE phase for experiments with *L. pneumophila* wild-type and its *csrA* mutant grown in CE MDM with either 6 mM [U-^13^C_3_]serine, 11 mM [U-^13^C_6_]glucose or 50 mM [U-^13^C_3_]glycerol as tracer.

**Table S9**: ^13^C-Excess (mol%) of protein-derived amino acids. diaminopimelic acid (DAP). poly-hydroxybutyrate (PHB). lactic acid (LACT) and stearic acid (STE) from experiments with *L. pneumophila* wild-type and its *csrA* mutant grown in CE MDM supplemented with 0.8 mM [1.2.3.4-^13^C_4_]palmitic acid.

**Table S10**: Relative fractions of isotopologues (mol%) of PHB and glutamic acid from *L. pneumophila* WT and its *csrA* mutant grown in CE MDM supplemented with 0.8 mM [1.2.3.4-^13^C_4_]palmitic acid.

**Table S11:** Composition of CE MDM

**Table S12.** Retention time and mass fragments of derivatized metabolites used for isotopologue calculations.

**Table S13.** Statistical analysis of oxygen consumption rate in *L. pneumophila* depicted in Figure 1

**Table S1**: **^13^C-Excess (mol%) of protein-derived amino acids, diaminopimelic acid (DAP), poly-hydroxybutyrate (PHB), mannose (Man), glucosamine (GlcN) and muramic acid (Mur) from experiments with *L. pneumophila* wild-type and its *csrA* mutant grown in CE MDM supplemented with 6 mM [U-^13^C_3_]serine*.**

| [U-^13^C_3_]serine | **WT E** | **WT PE** | ***ΔcsrA* E** | **Statistical significance of *ΔcsrA* E compared to WT E (p-value)**** | ***ΔcsrA* PE** | **Statistical significance of *ΔcsrA* PE compared to WT PE (p-value)**** |
| --- | --- | --- | --- | --- | --- | --- |
| **Ala** | 59.14% ± 2.60% | 63.22% ± 0.79% | 56.73% ± 0.62% | 0.069324 | 62.63% ± 1.20% | 0.337052 |
| **Asp** | 23.17% ± 1.98% | 25.87% ± 0.51% | 19.03% ± 0.84% | 0.002189 | 22.13% ± 0.07% | 0.000010 |
| **Glu** | 20.19% ± 1.03% | 24.77% ± 0.15% | 16.91% ± 0.19% | 0.000586 | 21.66% ± 0.85% | 0.000317 |
| **Gly** | 18.02% ± 0.70% | 16.73% ± 1.33% | 14.50% ± 0.15% | 0.000068 | 14.18% ± 1.51% | 0.011278 |
| **Ile** | 0.04% ± 0.01% | 0.06% ± 0.02% | 0.05% ± 0.02% |  | 0.06% ± 0.01% |  |
| **Leu** | 0.01% ± 0.00% | 0.01% ± 0.01% | 0.01% ± 0.01% |  | 0.01% ± 0.01% |  |
| **Lys** | 37.06% ± 2.48% | 40.66% ± 0.80% | 33.18% ± 1.07% | 0.009673 | 37.50% ± 0.48% | 0.000033 |
| **Phe** | 0.13% ± 0.07% | 0.12% ± 0.06% | 0.12% ± 0.06% |  | 0.13% ± 0.07% |  |
| **Pro** | 0.23% ± 0.04% | 0.35% ± 0.03% | 0.25% ± 0.04% |  | 0.28% ± 0.05% |  |
| **Ser** | 82.59% ± 0.61% | 82.69% ± 0.24% | 87.13% ± 8.61% | 0.253981 | 84.91% ± 3.21% | 0.152902 |
| **Tyr** | 0.07% ± 0.03% | 0.09% ± 0.02% | 0.11% ± 0.05% |  | 0.07% ± 0.06% |  |
| **Val** | 0.04% ± 0.03% | 0.03% ± 0.02% | 0.01% ± 0.01% |  | 0.04% ± 0.02% |  |
| **DAP** | 38.48% ± 1.44% | 43.51% ± 0.40% | 33.20% ± 1.84% | 0.000365 | 43.41% ± 0.78% | 0.778054 |
| **PHB** | 26.98% ± 2.98% | 28.84% ± 0.94% | 26.56% ± 1.70% | 0.771620 | 29.73% ± 0.87% | 0.118755 |
| **His** | 37.87% ± 2.99% | 36.48% ± 0.65% | 25.91% ± 0.72% | 0.000077 | 28.40% ± 2.46% | 0.000238 |
| **Man** | 24.95% ± 2.49% | 19.56% ± 1.63% | 13.13% ± 0.65% | 0.000049 | 12.99% ± 0.75% | 0.000079 |
| **GlcN** | 24.14% ± 2.19% | 20.48% ± 4.99% | 13.99% ± 3.22% | 0.000249 | 17.08% ± 3.99% | 0.260743 |
| **Mur** | 32.62% ± 6.07% | 31.96% ± 6.56% | 24.03% ± 2.07% | 0.024206 | 27.54% ± 4.41% | 0.242306 |

***** Cells were harvested at E phase (OD_600_ = 0.35) and PE phase (OD_600_ = 0.80). **Mean and standard deviation from two independent experiments. Statistical analysis was performed using two-tailed unpaired Student´s t-test for the analysis of differences between the wild-type and the *csrA* mutant at E and PE growth phase. Statistical significance is represented as p-value (*p < 0.05, ** p < 0.01 and ***p < 0.001).

**Table S2:** **Relative fractions of isotopologues (mol%) of protein-derived amino acids, diaminopimelic acid (DAP), poly-hydroxybutyrate (PHB), mannose (Man), glucosamine (GlcN) and muramic acid (Mur) from experiments with *L. pneumophila* wild-type and its *csrA* mutant grown in CE MDM supplemented with 6 mM [U-^13^C_3_]serine**.

| [U-^13^C_3_]serine: **WT E** | | | | | | | | | | | | |
| --- | --- | --- | --- | --- | --- | --- | --- | --- | --- | --- | --- | --- |
|  | **Ala** | **Asp** | **Glu** | **Gly** | **His** | **Lys** | **Ser** | **DAP** | **PHB** | **Man** | **GlcN** | **Mur** |
| **M+1*** | 4.55% ± 0.16% | 10.46% ± 0.39% | 7.29% ± 0.22% | 0.57% ± 0.06% | 17.23% ± 0.45% | 4.92% ± 0.32% | 1.75% ± 0.05% | 3.95% ± 0.26% | 5.82% ± 0.78% | 4.81% ± 0.18% | 5.71% ± 0.81% | 8.12% ± 3.16% |
| **M+2** | 4.40% ± 0.06% | 16.43% ± 0.86% | 17.77% ± 0.39% | 17.73% ± 0.71% | 8.03% ± 0.32% | 16.87% ± 0.37% | 2.59% ± 0.07% | 5.91% ± 0.65% | 28.53% ± 1.42% | 5.22% ± 0.30% | 6.34% ± 1.75% | 2.66% ± 2.33% |
| **M+3** | 54.68% ± 2.63% | 10.92% ± 1.08% | 8.17% ± 0.43% |  | 11.93% ± 0.27% | 20.33% ± 0.75% | 80.28% ± 0.59% | 28.43% ± 0.99% | 3.11% ± 0.78% | 15.90% ± 0.56% | 13.78% ± 2.33% | 20.05% ± 11.78% |
| **M+4** |  | 4.15% ± 0.68% | 5.59% ± 0.52% |  | 14.38% ± 0.40% | 12.62% ± 0.79% |  | 10.07% ± 0.99% | 8.92% ± 1.58% | 4.48% ± 0.29% | 3.91% ± 0.99% | 5.35% ± 3.71% |
| **M+5** |  |  | 2.24% ± 0.25% |  | 8.23% ± 0.86% | 10.06% ± 1.06% |  | 12.46% ± 0.50% |  | 3.67% ± 0.68% | 5.34% ± 2.84% | 2.76% ± 3.86% |
| **M+6** |  |  |  |  | 9.91% ± 2.16% | 3.66% ± 0.63% |  | 7.60% ± 0.26% |  | 8.41% ± 2.00% | 7.13% ± 1.77% | 14.48% ± 4.97% |
| **M+7** |  |  |  |  |  |  |  | 2.87% ± 0.22% |  |  |  |  |
| [U-^13^C_3_]serine: **WT PE** | | | | | | | | | | | | |
|  | **Ala** | **Asp** | **Glu** | **Gly** | **His** | **Lys** | **Ser** | **DAP** | **PHB** | **Man** | **GlcN** | **Mur** |
| **M+1** | 6.47% ± 0.28% | 14.48% ± 0.58% | 12.66% ± 0.36% | 0.56% ± 0.12% | 27.92% ± 1.97% | 7.40% ± 0.34% | 4.99% ±1.12% | 7.77% ± 0.53% | 9.18% ± 0.28% | 6.53% ± 1.46% | 11.71% ± 13.14% | 13.11% ± 9.66% |
| **M+2** | 4.87% ± 0.11% | 18.18% ± 0.30% | 21.62% ± 0.14% | 16.44% ± 1.28% | 9.63% ± 0.24% | 19.42% ± 0.48% | 3.26% ± 0.19% | 8.38% ± 0.34% | 32.33% ± 0.73% | 5.71% ± 0.47% | 8.29% ± 5.64% | 12.85% ± 10.80% |
| **M+3** | 57.82% ± 0.77% | 11.99% ± 0.45% | 10.72% ± 0.18% |  | 13.27% ± 0.20% | 23.05% ± 0.87% | 78.85% ± 0.28% | 30.52% ± 0.32% | 3.62% ± 0.22% | 16.44% ± 1.06% | 9.36% ± 5.47% | 6.68% ± 7.67% |
| **M+4** |  | 4.17% ± 0.12% | 5.96% ± 0.27% |  | 15.25% ± 0.43% | 14.11% ± 0.26% |  | 14.03% ± 0.27% | 7.66% ± 0.52% | 3.29% ± 0.81% | 6.96% ± 5.43% | 14.87% ± 9.49% |
| **M+5** |  |  | 2.38% ± 0.10% |  | 6.64% ± 0.19% | 10.08% ± 0.13% |  | 12.98% ± 0.51% |  | 2.27% ± 0.26% | 6.36% ± 5.82% | 4.94% ± 8.46% |
| **M+6** |  |  |  |  | 6.28% ± 0.17% | 3.63% ± 0.09% |  | 8.05% ± 0.26% |  | 4.26% ± 0.92% | 1.15% ± 1.00% | 8.12% ± 7.87% |
| **M+7** |  |  |  |  |  |  |  | 2.74% ± 0.16% |  |  |  |  |
| [U-^13^C_3_]serine: ***ΔcsrA* E** | | | | | | | | | | | | |
|  | **Ala** | **Asp** | **Glu** | **Gly** | **His** | **Lys** | **Ser** | **DAP** | **PHB** | **Man** | **GlcN** | **Mur** |
| **M+1** | 5.08% ± 0.71% | 10.94% ± 0.18% | 7.80% ± 0.65% | 0.37% ± 0.06% | 30.77% ± 1.77% | 6.12% ± 0.82% | 1.81% ± 0.73% | 4.95% ± 1.11% | 5.49% ± 0.34% | 4.67% ± 0.37% | 6.30% ± 1.20% | 4.98% ± 3.04% |
| **M+2** | 4.04% ± 0.35% | 14.94% ± 0.51% | 17.31% ± 0.16% | 14.31% ± 0.14% | 6.84% ± 0.44% | 17.66% ± 0.38% | 2.67% ± 0.21% | 5.92% ± 0.63% | 30.61% ± 1.81% | 3.92% ± 0.15% | 4.59% ± 0.92% | 6.82% ± 2.94% |
| **M+3** | 52.34% ± 1.05% | 8.59% ± 0.58% | 6.55% ± 0.20% |  | 8.79% ± 0.24% | 20.55% ± 0.72% | 84.74% ± 8.71% | 29.88% ± 1.80% | 2.62% ± 0.11% | 11.99% ± 0.93% | 10.72% ± 1.85% | 16.00% ± 1.93% |
| **M+4** |  | 2.38% ± 0.22% | 4.05% ± 0.12% |  | 12.46% ± 0.77% | 11.18% ± 0.46% |  | 8.60% ± 0.60% | 7.92% ± 0.88% | 2.00% ± 0.19% | 2.63% ± 1.51% | 8.24% ± 2.93% |
| **M+5** |  |  | 1.25% ± 0.11% |  | 3.48% ± 0.52% | 7.77% ± 0.56% |  | 9.92% ± 0.59% |  | 1.32% ± 0.27% | 2.39% ± 1.11% | 1.14% ± 1.27% |
| **M+6** |  |  |  |  | 2.90% ± 0.20% | 2.07% ± 0.21% |  | 5.27% ± 0.37% |  | 2.61% ± 0.32% | 2.30% ± 1.01% | 6.49% ± 1.65% |
| **M+7** |  |  |  |  |  |  |  | 1.47% ± 0.31% |  |  |  |  |
| [U-^13^C_3_]serine: ***ΔcsrA* PE** | | | | | | | | | | | | |
|  | **Ala** | **Asp** | **Glu** | **Gly** | **His** | **Lys** | **Ser** | **DAP** | **PHB** | **Man** | **GlcN** | **Mur** |
| **M+1** | 5.58% ± 1.02% | 13.18% ± 0.86% | 11.04% ± 1.29% | 0.43% ± 0.07% | 36.30% ± 0.18% | 6.68% ± 1.10% | 3.11% ± 0.53% | 5.27% ± 1.06% | 6.76% ± 0.77% | 4.94% ± 0.92% | 10.25% ± 5.20% | 11.72% ± 4.79% |
| **M+2** | 4.25% ± 0.55% | 16.81% ± 0.39% | 20.35% ± 0.43% | 13.97% ± 1.49% | 7.73% ± 1.06% | 19.05% ± 0.82% | 2.82% ± 0.25% | 6.54% ± 1.21% | 34.25% ± 0.49% | 4.10% ± 0.39% | 7.52% ± 3.06% | 3.76% ± 3.33% |
| **M+3** | 57.93% ± 0.53% | 10.09% ± 0.31% | 9.01% ± 0.61% |  | 9.55% ± 0.85% | 22.72% ± 0.53% | 81.99% ± 2.88% | 32.62% ± 1.71% | 2.96% ± 0.13% | 12.65% ± 0.20% | 10.03% ± 3.83% | 21.00% ± 5.35% |
| **M+4** |  | 2.87% ± 0.18% | 5.19% ± 0.10% |  | 13.13% ± 1.05% | 12.86% ± 0.23% |  | 13.19% ± 0.62% | 8.69% ± 0.78% | 1.79% ± 0.38% | 4.84% ± 4.41% | 5.54% ± 3.91% |
| **M+5** |  |  | 1.75% ± 0.06% |  | 3.70% ± 0.48% | 8.95% ± 0.26% |  | 13.63% ± 0.38% |  | 1.16% ± 0.35% | 1.94% ± 1.29% | 2.57% ± 0.99% |
| **M+6** |  |  |  |  | 3.16% ± 0.59% | 2.65% ± 0.08% |  | 8.22% ± 0.69% |  | 2.32% ± 0.33% | 3.00% ± 2.65% | 8.00% ± 4.85% |
| **M+7** |  |  |  |  |  |  |  | 2.49% ± 0.29% |  |  |  |  |

**°** M+X represents the mass of the unlabeled metabolite plus X labeled ^13^C-atoms. Shown are mean and standard deviations from two independent experiments.

**Table S3**: **^13^C-Excess (mol%) of protein-derived amino acids, diaminopimelic acid (DAP), poly-hydroxybutyrate (PHB), mannose (Man), glucosamine (GlcN) and muramic acid (Mur) from experiments with *L. pneumophila* wild-type and its *csrA* mutant grown in CE MDM supplemented with 11 mM [U-^13^C_6_]glucose*.**

| [U-^13^C_6_]glucose | **WT E** | **WT PE** | ***ΔcsrA* E** | **Statistical significance of *ΔcsrA* E compared to WT E (p-value)**** | ***ΔcsrA* PE** | **Statistical significance of *ΔcsrA* PE compared to WT PE (p-value)**** |
| --- | --- | --- | --- | --- | --- | --- |
| **Ala** | 3.95% ± 0.23% | 6.30% ± 0.94% | 4.15% ± 0.23% | 0.166662 | 4.80% ± 0.10% | 0.011655 |
| **Asp** | 1.57% ± 0.20% | 3.13% ± 0.42% | 1.53% ± 0.26% | 0.797153 | 2.09% ± 0.07% | 0.001916 |
| **Glu** | 1.50% ± 0.04% | 2.77% ± 0.46% | 1.41% ± 0.61% | 0.288042 | 1.86% ± 0.07% | 0.004714 |
| **Gly** | 0.10% ± 0.09% | 0.11% ± 0.04% | 0.07% ± 0.05% |  | 0.12% ± 0.09% |  |
| **Ile** | 0.06% ± 0.04% | 0.05% ± 0.02% | 0.06% ± 0.01% |  | 0.06% ± 0.03% |  |
| **Leu** | 0.02% ± 0.02% | 0.01% ± 0.01% | 0.01% ± 0.01% |  | 0.01% ± 0.00% |  |
| **Lys** | 2.31% ± 0.17% | 3.98% ± 0.46% | 2.55% ± 0.18% | 0.038413 | 3.19% ± 0.04% | 0.008491 |
| **Phe** | 0.09% ± 0.04% | 0.12% ± 0.01% | 0.09% ± 0.04% |  | 0.08% ± 0.02% |  |
| **Pro** | 0.22% ± 0.03% | 0.24% ± 0.04% | 0.22% ± 0.04% |  | 0.24% ± 0.04% |  |
| **Ser** | 0.22% ± 0.08% | 0.36% ± 0.20% | 0.19% ± 0.06% |  | 0.16% ± 0.03% |  |
| **Tyr** | 0.10% ± 0.03% | 0.08% ± 0.02% | 0.06% ± 0.02% |  | 0.10% ± 0.05% |  |
| **Val** | 0.04% ± 0.03% | 0.02% ± 0.02% | 0.02% ± 0.01% |  | 0.05% ± 0.03% |  |
| **DAP** | 3.21% ± 058% | 5.19% ± 1.33% | 3.42% ± 0.27% | 0.442887 | 3.76% ± 0.12% | 0.046997 |
| **PHB** | 1.76% ± 0.67% | 3.17% ± 1.35% | 1.72% ± 0.32% | 0.892044 | 1.99% ± 0.28% | 0.090576 |
| **His** | 19.93% ± 0.67% | 26.97% ± 2.05% | 23.62% ± 2.53% | 0.013506 | 28.59% ± 0.14% | 0.110587 |
| **Man** | 46.94% ± 9.19% | 61.03% ± 9.50% | 61.08% ± 2.38% | 0.015799 | 60.70% ± 13.10% | 0.964660 |
| **GlcN** | 31.62% ± 4.59% | 36.68% ± 4.53% | 38.91% ± 2.25% | 0.015213 | 34.47% ± 1.58% | 0.342160 |
| **Mur** | 16.58% ± 2.20% | 28.51% ± 1.34% | 21.57% ± 11.48% | 0.384274 | 25.05% ± 2.77% | 0.040131 |

***** Cells were harvested at E phase (OD_600_ = 0.35) and PE phase (OD_600_ = 0.80). **Mean and standard deviations from two independent experiments. Statistical analysis was performed using two-tailed unpaired Student´s t-test for the analysis of differences between the wild-type and the *csrA* mutant at E and PE growth phase. Statistical significance is represented as p-value (*p < 0.05, ** p < 0.01 and ***p < 0.001).

**Table S4:** **Relative fractions of isotopologues (mol%) of protein-derived amino acids, diaminopimelic acid (DAP), poly-hydroxybutyrate (PHB), mannose (Man), glucosamine (GlcN) and muramic acid (Mur) from experiments with *L. pneumophila* wild-type and its *csrA* mutant grown in CE MDM supplemented with 11 mM [U-^13^C_6_]glucose.**

| [U-^13^C_6_]glucose: **WT E** | | | | | | | | | | | | |
| --- | --- | --- | --- | --- | --- | --- | --- | --- | --- | --- | --- | --- |
|  | **Ala** | **Asp** | **Glu** | **Gly** | **His** | **Lys** | **Ser** | **DAP** | **PHB** | **Man** | **GlcN** | **Mur** |
| **M+1*** | 0.52% ± 0.12% | 1.98% ± 0.52% | 1.65% ± 0.36% |  | 5.72% ± 0.31% | 2.31% ± 0.13% |  | 2.47% ± 1.22% | 1.23% ± 0.71% | 2.42% ± 1.05% | 4.98% ± 0.96% | 13.34% ± 1.67% |
| **M+2** | 0.37% ± 0.13% | 1.15% ± 0.10% | 2.43% ± 0.16% |  | 13.44% ± 0.32% | 2.48% ± 0.44% |  | 2.08% ± 0.59% | 2.67% ± 0.98% | 2.25% ± 1.61% | 5.49% ± 0.72% | 2.90% ± 1.53% |
| **M+3** | 3.53% ± 0.20% | 0.65% ± 0.06% | 0.25% ± 0.03% |  | 9.15% ± 0.52% | 2.19% ± 0.14% |  | 4.49% ± 0.74% | 0.07% ± 0.16% | 8.41% ± 4.86% | 12.01% ± 2.13% | 7.81% ± 2.76% |
| **M+4** |  | 0.01% ± 0.01% | 0.02% ± 0.02% |  | 3.93% ± 0.08% | 0.00% ± 0.00% |  | 0.34% ± 0.15% | 0.06% ± 0.05% | 2.12% ± 1.19% | 3.60% ± 0.32% | 2.28% ± 2.40% |
| **M+5** |  |  | 0.03% ± 0.01% |  | 8.75% ± 1.00% | 0.00% ± 0.00% |  | 0.13% ± 0.08% |  | 2.73% ± 0.62% | 4.85% ± 0.91% | 0.50% ± 0.78% |
| **M+6** |  |  |  |  | 0.00% ± 0.00% | 0.00% ± 0.00% |  | 0.04% ± 0.04% |  | 37.89% ± 13.91% | 16.51% ± 2.74% | 7.55% ± 1.40% |
| **M+7** |  |  |  |  |  |  |  | 0.01% ± 0.03% |  |  |  |  |
| [U-^13^C_6_]glucose: **WT PE** | | | | | | | | | | | | |
|  | **Ala** | **Asp** | **Glu** | **Gly** | **His** | **Lys** | **Ser** | **DAP** | **PHB** | **Man** | **GlcN** | **Mur** |
| **M+1** | 1.08% ± 0.29% | 4.02% ± 0.30% | 3.71% ± 0.45% |  | 6.48% ± 0.20% | 4.45% ± 0.46% |  | 4.84% ± 0.95% | 1.82% ± 0.76% | 3.58% ± 1.39% | 8.17% ± 0.35% | 13.11% ± 3.76% |
| **M+2** | 0.67% ± 0.22% | 2.30% ± 0.33% | 4.01% ± 0.64% |  | 16.58% ± 1.00% | 4.30% ± 0.51% |  | 3.65% ± 0.89% | 5.06% ± 2.09% | 3.96% ± 1.51% | 7.56% ± 0.18% | 8.07% ± 0.73% |
| **M+3** | 5.50% ± 0.82% | 1.21% ± 0.21% | 0.50% ± 0.17% |  | 11.21% ± 1.07% | 3.54% ± 0.37% |  | 6.28% ± 1.64% | 0.07% ± 0.09% | 14.26% ± 7.23% | 14.27% ± 1.72% | 11.08% ± 2.99% |
| **M+4** |  | 0.06% ± 0.04% | 0.08% ± 0.05% |  | 5.06% ± 0.47% | 0.02% ± 0.03% |  | 0.73% ± 0.26% | 0.13% ± 0.07% | 3.92% ± 1.86% | 4.53% ± 0.38% | 9.29% ± 0.83% |
| **M+5** |  |  | 0.06% ± 0.02% |  | 13.67% ± 1.03% | 0.03% ± 0.04% |  | 0.31% ± 0.16% |  | 4.01% ± 0.53% | 6.02% ± 0.77% | 1.31% ± 0.39% |
| **M+6** |  |  |  |  | 0.00% ± 0.00% | 0.00% ± 0.00% |  | 0.13% ± 0.07% |  | 46.03% ± 16.40% | 17.63% ± 3.24% | 10.81% ± 0.46% |
| **M+7** |  |  |  |  |  |  |  | 0.01% ± 0.01% |  |  |  |  |
| [U-^13^C_6_]glucose: ***ΔcsrA* E** | | | | | | | | | | | | |
|  | **Ala** | **Asp** | **Glu** | **Gly** | **His** | **Lys** | **Ser** | **DAP** | **PHB** | **Man** | **GlcN** | **Mur** |
| **M+1** | 0.65% ± 0.24% | 2.19% ± 0.57% | 1.83% ± 0.40% |  | 5.35% ± 0.26% | 2.61% ± 0.24% |  | 2.63% ± 0.83% | 1.16% ± 0.57% | 1.98% ± 0.67% | 4.10% ± 0.90% | 5.64% ± 3.82% |
| **M+2** | 0.33% ± 0.05% | 1.15% ± 0.21% | 2.21% ± 0.22% |  | 14.78% ± 1.30% | 2.72% ± 0.23% |  | 2.25% ± 0.56% | 2.74% ± 0.43% | 2.78% ± 0.59% | 5.32% ± 0.56% | 3.56% ± 2.14% |
| **M+3** | 3.71% ± 0.19% | 0.54% ± 0.09% | 0.21% ± 0.07% |  | 10.12% ± 0.65% | 2.41% ± 0.22% |  | 4.60% ± 0.20 | 0.01% ± 0.01% | 9.07% ± 2.37% | 12.59% ± 0.61% | 6.29% ± 2.42% |
| **M+4** |  | 0.00% ± 0.01% | 0.01% ± 0.01% |  | 5.08% ± 0.39% | 0.00% ± 0.00% |  | 0.44% ± 0.12%% | 0.06% ± 0.02% | 3.26% ± 0.94% | 4.76% ± 0.36% | 5.46% ± 5.03% |
| **M+5** |  |  | 0.03% ± 0.00% |  | 11.22% ± 1.81% | 0.00% ± 0.00% |  | 0.17% ± 0.08% |  | 3.46% ± 0.37% | 5.65% ± 0.82%% | 0.98% ± 1.22% |
| **M+6** |  |  |  |  | 0.00% ± 0.00% | 0.00% ± 0.00% |  | 0.07% ± 0.05% |  | 50.23% ± 0.55% | 22.28% ± 1.23% | 11.85% ± 8.81% |
| **M+7** |  |  |  |  |  |  |  | 0.01% ± 0.01% |  |  |  |  |
| [U-^13^C_6_]glucose: ***ΔcsrA* PE** | | | | | | | | | | | | |
|  | **Ala** | **Asp** | **Glu** | **Gly** | **His** | **Lys** | **Ser** | **DAP** | **PHB** | **Man** | **GlcN** | **Mur** |
| **M+1** | 0.76% ± 0.14% | 2.75% ± 0.25% | 2.72% ± 0.27% |  | 5.58% ± 0.21% | 3.62% ± 0.21% |  | 3.09% ± 0.51% | 1.24% ± 0.36% | 2.35% ± 1.01% | 7.68% ± 0.23% | 9.78% ± 1.52% |
| **M+2** | 0.44% ± 0.06% | 1.57% ± 0.06% | 2.68% ± 0.12% |  | 17.18% ± 0.76% | 3.45% ± 0.09% |  | 2.69% ± 0.31% | 3.30% ± 0.43% | 2.78% ± 0.94% | 6.65% ± 0.24% | 7.55% ± 1.61% |
| **M+3** | 4.26% ± 0.06% | 0.81% ± 0.07% | 0.29% ± 0.03% |  | 11.56% ± 0.49% | 2.88% ± 0.11% |  | 4.90% ± 0.25% | 0.01% ± 0.01% | 9.13% ± 2.49% | 11.99% ± 0.25% | 7.45% ± 1.19% |
| **M+4** |  | 0.01% ± 0.02% | 0.03% ± 0.02% |  | 5.92% ± 0.22% | 0.00% ± 0.00% |  | 0.39% ± 0.10% | 0.03% ± 0.04% | 2.55% ± 0.43% | 4.28% ± 0.14% | 6.80% ± 1.65% |
| **M+5** |  |  | 0.04% ± 0.00% |  | 14.65% ± 0.53% | 0.00% ± 0.00% |  | 0.18% ± 0.13% |  | 3.63% ± 0.41% | 5.44% ± 0.71% | 0.68% ± 0.78% |
| **M+6** |  |  |  |  | 0.00% ± 0.00% | 0.00% ± 0.00% |  | 0.09% ± 0.04% |  | 50.09% ± 16.03% | 17.59% ± 1.22% | 12.08% ± 2.48% |
| **M+7** |  |  |  |  |  |  |  | 0.02% ± 0.03% |  |  |  |  |

***** M+X represents the mass of the unlabeled metabolite plus X labeled ^13^C-atoms. Shown are mean and standard deviation from two independent experiments.

| [U-^13^C_3_]glycerol | **WT E** | **WT PE** | ***ΔcsrA* E** | **Statistical significance of *ΔcsrA* E compared to WT E (p-value)**** | ***ΔcsrA* PE** | **Statistical significance of *ΔcsrA* PE compared to WT PE (p-value)**** |
| --- | --- | --- | --- | --- | --- | --- |
| **Ala** | 0.51% ± 0.11% | 0.83% ± 0.27% | 1.11% ± 0.05% | 0.0000058 | 1.42% ± 0.16% | 0.00173557 |
| **Asp** | 0.24% ± 0.07% | 0.33% ± 0.23% | 0.49% ± 0.05% | 0.0000960 | 0.63% ± 0.09% | 0.02279813 |
| **Glu** | 0.27% ± 0.05% | 0.48% ± 0.19% | 0.48% ± 0.03% | 0.0000078 | 0.77% ± 0.12% | 0.01419635 |
| **Gly** | 0.08% ± 0.07% | 0.03% ± 0.06% | 0.08% ± 0.04% |  | 0.08% ± 0.08% |  |
| **Ile** | 0.08% ± 0.04% | 0.07% ± 0.05% | 0.09% ± 0.04% |  | 0.07% ± 0.05% |  |
| **Leu** | 0.06% ± 0.06% | 0.06% ± 0.06% | 0.06% ± 0.05% |  | 0.04% ± 0.04% |  |
| **Lys** | 0.33% ± 0.06% | 0.57% ± 0.18% | 0.71% ± 0.04% | 0.0000005 | 0.94% ± 0.13% | 0.00228983 |
| **Phe** | 0.16% ± 0.11% | 0.16% ± 0.09% | 0.15% ± 0.08% |  | 0.16% ± 0.08% |  |
| **Pro** | 0.31% ± 0.05% | 0.26% ± 0.07% | 0.26% ± 0.07% |  | 0.27% ± 0.14% |  |
| **Ser** | 0.14% ± 0.04% | 0.12% ± 0.06% | 0.16% ± 0.06% |  | 0.13% ± 0.04% |  |
| **Tyr** | 0.12% ± 0.05% | 0.11% ± 0.06% | 0.11% ± 0.03% |  | 0.13% ± 0.03% |  |
| **Val** | 0.04% ± 0.03% | 0.04% ± 0.06% | 0.04% ± 0.04% |  | 0.05% ± 0.03% |  |
| **DAP** | 0.55% ± 0.11% | 1.08% ± 0.11% | 0.98% ± 0.08% | 0.0000336 | 1.56% ± 0.14% | 0.00011700 |
| **PHB** | 0.57% ± 0.23% | 0.49% ± 0.17% | 0.49% ± 0.19% | 0.5492660 | 0.68% ± 0.19% | 0.09496287 |
| **His** | 5.45% ± 0.90% | 6.40% ± 1.08% | 13.79% ± 1.35% | 0.0000005 | 13.99% ± 0.03% | 0.00001195 |
| **Man** | 4.22% ± 0.98% | 6.27% ± 1.24% | 12.70% ± 0.56% | 0.0000002 | 16.00% ± 0.79% | 0.00000044 |
| **GlcN** | 6.56% ± 1.42% | 18.35% ± 2.44% | 13.44% ± 2.03% | 0.0001560 | 23.35% ± 5.19% | 0.09260024 |
| **Mur** | 8.61% ± 1.58% | 20.39% ± 5.24% | 19.65% ± 5.43% | 0.0047552 | 28.21% ± 5.14% | 0.03847291 |

**Table S5**: **^13^C-Excess (mol%) of protein-derived amino acids, diaminopimelic acid (DAP), poly-hydroxybutyrate (PHB), mannose (Man), glucosamine (GlcN) and muramic acid (Mur) from experiments with *L. pneumophila* wild-type and its *csrA* mutant grown in CE MDM supplemented with 50 mM [U-^13^C_3_]glycerol*.**

* Cells were harvested at E phase (OD_600_ = 0.35) and PE phase (OD_600_ = 0.80). **Mean and standard deviation from two independent experiments. Statistical analysis was performed using two-tailed unpaired Student´s t-test for the analysis of differences between the wild-type and the *csrA* mutant at E and PE growth phase. Statistical significance is represented as p-value (*p < 0.05, ** p < 0.01 and ***p < 0.001).

**Table S6:** **Relative fractions of isotopologues (mol%) of protein-derived amino acids, diaminopimelic acid (DAP), poly-hydroxybutyrate (PHB), mannose (Man), glucosamine (GlcN) and muramic acid (Mur) from experiments with *L. pneumophila* wild-type and its *csrA* mutant grown in CE MDM supplemented with 50 mM [U-^13^C_3_]glycerol.**

| [U-^13^C_3_]glycerol: **WT E** | | | | | | | | | | | | |
| --- | --- | --- | --- | --- | --- | --- | --- | --- | --- | --- | --- | --- |
|  | **Ala** | **Asp** | **Glu** | **Gly** | **His** | **Lys** | **Ser** | **DAP** | **PHB** | **Man** | **GlcN** | **Mur** |
| **M+1*** | 0,43% ± 0,21% |  | 0,36% ± 0,24% |  | 3,53% ± 0,75% | 0,23% ± 0,25% |  | 0,55% ± 0,62% | 0,71% ± 0,25% | 2,37% ± 0,76% | 4,15% ± 0,74% | 2,15% ± 3,07% |
| **M+2** | 0,04% ± 0,04% |  | 0,47% ± 0,08% |  | 4,17% ± 0,67% | 0,11% ± 0,13% |  | 0,36% ± 0,31% | 0,74% ± 0,48% | 1,79% ± 0,40% | 2,15% ± 0,52% | 2,95% ± 2,79% |
| **M+3** | 0,35% ± 0,03% |  | 0,01% ± 0,03% |  | 4,20% ± 0,80% | 0,51% ± 0,04% |  | 0,66% ± 0,22% | 0,01% ± 0,03% | 5,41% ± 1,38% | 4,49% ± 0,59% | 3,77% ± 2,17% |
| **M+4** |  |  | 0,00% ± 0,00% |  | 0,62% ± 0,17% | 0,00% ± 0,00% |  | 0,05% ± 0,06% | 0,01% ± 0,02% | 0,16% ± 0,08% | 0,74% ± 0,65% | 5,70% ± 2,05% |
| **M+5** |  |  | 0,00% ± 0,01% |  | 1,16% ± 0,09% | 0,00% ± 0,00% |  | 0,06% ± 0,12% |  | 0,18% ± 0,04% | 1,58% ± 0,73% | 0,59% ± 0,97% |
| **M+6** |  |  |  |  | 0,00% ± 0,00% | 0,00% ± 0,00% |  | 0,01% ± 0,02% |  | 0,26% ± 0,08% | 1,09% ± 0,71% | 1,10% ± 0,88% |
| **M+7** |  |  |  |  |  |  |  | 0,01% ± 0,02% |  |  |  |  |
| [U-^13^C_3_]glycerol: **WT PE** | | | | | | | | | | | | |
|  | **Ala** | **Asp** | **Glu** | **Gly** | **His** | **Lys** | **Ser** | **DAP** | **PHB** | **Man** | **GlcN** | **Mur** |
| **M+1** | 0,52% ± 0,35% |  | 0,67% ± 0,46% |  | 3,98% ± 0,92% | 1,01% ± 0,66% |  | 1,44% ± 0,94% | 0,56% ± 0,39% | 2,95% ± 1,22% | 6,54% ± 1,18% | 6,22% ± 3,13% |
| **M+2** | 0,07% ± 0,10% |  | 0,81% ± 0,28% |  | 4,80% ± 0,75% | 0,20% ± 0,14% |  | 0,80% ± 0,39% | 0,68% ± 0,16% | 2,63% ± 0,31% | 5,67% ± 0,59% | 7,14% ± 3,36% |
| **M+3** | 0,60% ± 0,12% |  | 0,02% ± 0,02% |  | 5,14% ± 1,09% | 0,67% ± 0,09% |  | 1,39% ± 0,20% | 0,01% ± 0,01% | 7,41% ± 1,33% | 9,44% ± 0,98% | 8,77% ± 1,61% |
| **M+4** |  |  | 0,00% ± 0,00% |  | 0,69% ± 0,12% | 0,00% ± 0,00% |  | 0,07% ± 0,07% | 0,00% ± 0,00% | 0,42% ± 0,28% | 2,84% ± 0,32% | 7,78% ± 3,13% |
| **M+5** |  |  | 0,01% ± 0,01% |  | 1,34% ± 0,14% | 0,00% ± 0,00% |  | 0,00% ± 0,00% |  | 0,35% ± 0,28% | 5,04% ± 1,35% | 1,62% ± 1,32% |
| **M+6** |  |  |  |  | 0,00% ± 0,00% | 0,00% ± 0,00% |  | 0,00% ± 0,01% |  | 0,62% ± 0,37% | 4,56% ± 0,96% | 6,05% ± 2,55% |
| **M+7** |  |  |  |  |  |  |  | 0,01% ± 0,01% |  |  |  |  |
| [U-^13^C_3_]glycerol: ***ΔcsrA* E** | | | | | | | | | | | | |
|  | **Ala** | **Asp** | **Glu** | **Gly** | **His** | **Lys** | **Ser** | **DAP** | **PHB** | **Man** | **GlcN** | **Mur** |
| **M+1** | 0,48% ± 0,13% | 1,37% ± 0,35% | 0,82% ± 0,13% |  | 6,90% ± 0,47% | 0,98% ± 0,39% |  | 1,24% ± 1,00% | 0,57% ± 0,47% | 4,81% ± 0,26% | 5,48% ± 1,68% | 5,80% ± 7,94% |
| **M+2** | 0,19% ± 0,10% | 0,01% ± 0,02% | 0,71% ± 0,11% |  | 10,25% ± 0,81% | 0,53% ± 0,19% |  | 0,92% ± 0,36% | 0,69% ± 0,16% | 4,79% ± 0,41% | 5,18% ± 3,98% | 6,53% ± 5,75% |
| **M+3** | 0,83% ± 0,10% | 0,19% ± 0,06% | 0,04% ± 0,05% |  | 9,39% ± 0,88% | 0,74% ± 0,09% |  | 1,19% ± 0,30% | 0,00% ± 0,00% | 13,47% ± 0,52% | 10,79% ± 2,32% | 8,78% ± 3,50% |
| **M+4** |  | 0,00% ± 0,00% | 0,00% ± 0,00% |  | 1,80% ± 0,23% | 0,00% ± 0,00% |  | 0,01% ± 0,02% | 0,00% ± 0,00% | 1,23% ± 0,12% | 1,73% ± 2,28% | 10,70% ± 5,92% |
| **M+5** |  |  | 0,01% ± 0,01% |  | 4,00% ± 0,52% | 0,00% ± 0,00% |  | 0,02% ± 0,04% |  | 0,79% ± 0,08% | 2,07% ± 0,91% | 2,09% ± 2,67% |
| **M+6** |  |  |  |  | 0,00% ± 0,00% | 0,00% ± 0,00% |  | 0,00% ± 0,00% |  | 2,09% ± 0,21% | 2,52% ± 0,96% | 3,24% ± 3,76% |
| **M+7** |  |  |  |  |  |  |  | 0,01% ± 0,03% |  |  |  |  |
| [U-^13^C_3_]glycerol: ***ΔcsrA* PE** | | | | | | | | | | | | |
|  | **Ala** | **Asp** | **Glu** | **Gly** | **His** | **Lys** | **Ser** | **DAP** | **PHB** | **Man** | **GlcN** | **Mur** |
| **M+1** | 0,56% ± 0,16% | 1,45% ± 0,45% | 1,33% ± 0,35% |  | 7,10% ± 0,16% | 1,53% ± 0,39% |  | 1,74% ± 0,82% | 0,73% ± 0,16% | 5,61% ± 0,47% | 7,22% ± 1,15% | 4,49% ± 1,73% |
| **M+2** | 0,23% ± 0,10% | 0,08% ± 0,08% | 1,13% ± 0,14% |  | 10,38% ± 0,21% | 0,68% ± 0,13% |  | 1,34% ± 0,31% | 0,98% ± 0,33% | 4,77% ± 0,90% | 6,62% ± 0,50% | 6,72% ± 1,92% |
| **M+3** | 1,08% ± 0,08% | 0,30% ± 0,04% | 0,06% ± 0,05% |  | 9,81% ± 0,29% | 0,92% ± 0,11% |  | 2,00% ± 0,21% | 0,00% ± 0,00% | 15,72% ± 0,93% | 13,27% ± 1,09% | 12,87% ± 2,07% |
| **M+4** |  | 0,00% ± 0,00% | 0,00% ± 0,00% |  | 1,76% ± 0,11% | 0,00% ± 0,00% |  | 0,07% ± 0,07% | 0,00% ± 0,00% | 1,93% ± 0,31% | 3,01% ± 0,89% | 14,12% ± 2,70% |
| **M+5** |  |  | 0,01% ± 0,00% |  | 3,93% ± 0,14% | 0,00% ± 0,00% |  | 0,03% ± 0,03% |  | 1,30% ± 0,18% | 5,68% ± 1,85% | 1,85% ± 1,44% |
| **M+6** |  |  |  |  | 0,00% ± 0,00% | 0,00% ± 0,00% |  | 0,00% ± 0,00% |  | 3,24% ± 0,47% | 6,57% ± 3,11% | 7,83% ± 5,26% |
| **M+7** |  |  |  |  |  |  |  | 0,00% ± 0,01% |  |  |  |  |

***** M+X represents the mass of the unlabeled metabolite plus X labeled ^13^C-atoms. Shown are mean and standard deviation from two independent experiments.

**Table S7:** **Ratio of ^13^C-excess in histidine to alanine calculated for E phase and PE phase for experiments with *L. pneumophila* wild-type and its *csrA* mutant grown in CE MDM with either 6 mM [U-^13^C_3_]serine, 11 mM [U-^13^C_6_]glucose or 50 mM [U-^13^C_3_]glycerol as tracers.**

| **Ratio**: ^13^C-Excess (mol%) **His**/^13^C-Excess (mol %) **Ala** | | | | | | |
| --- | --- | --- | --- | --- | --- | --- |
|  | | | | | | |
|  | **WT E** | **WT PE** | ***ΔcsrA* E** | **Statistical significance of ΔCsrA E compared to WT E (p-value)** | ***ΔcsrA* PE** | **Statistical significance of ΔCsrA E compared to WT E (p-value)*** |
| 6 mM [U-^13^C_3_]serine | 0.64 ± 0.05 | 0.58 ± 0.01 | 0.46 ± 0.01 | 3.67548E-22 | 0.45 ± 0.04 | 3.1765E-22 |
| 11 mM [U-^13^C_6_]glucose | 5.05 ± 0.34 | 4.36 ± 0.68 | 5.70 ± 0.63 | 1.42454E-06 | 5.95 ± 0.12 | 2.7E-16 |
| 50 mM [U-^13^C_3_]glycerol | 11.07 ± 2.73 | 8.61 ± 3.29 | 12.42 ± 1.26 | 0.009840751 | 9.98 ± 1.04 | 0.02089581 |

***** Mean values were calculated from matrix calculations (resulting in 36 data points) for possible **His/Ala** ratios with six data sets for His and Ala, respectively (2 biological and 2 x 3 technical replicates). Standard deviation was calculated from the resulting 36 **His/Ala** ratios. Statistical analysis was performed using two-tailed unpaired Student´s t-test for the analysis of differences between the wild-type and the *csrA* mutant at E and PE growth phase. Statistical significance is represented as p-value (*p < 0.05, ** p < 0.01 and ***p < 0.001).

**Table S8:** **Ratio of ^13^C-excess in histidine to glutamine calculated for E phase and PE phase for experiments with *L. pneumophila* wild-type and its *csrA* mutant grown in CE MDM with either 6 mM [U-^13^C_3_]serine, 11 mM [U-^13^C_6_]glucose or 50 mM [U-^13^C_3_]glycerol as tracer.**

| **Ratio**: ^13^C-Excess (mol%) **His**/^13^C-Excess (mol %) **Glu** | | | | | | |
| --- | --- | --- | --- | --- | --- | --- |
|  | | | | | | |
|  | **WT E** | **WT PE** | ***ΔcsrA* E** | **Statistical significance of ΔCsrA E compared to WT E (p-value)** | ***ΔcsrA* PE** | **Statistical significance of ΔCsrA E compared to WT E (p-value)*** |
| 6 mM [U-^13^C_3_]serine | 1.88 ± 0.16 | 1.47 ± 0.03 | 1.53 ± 0.04 | 3.39821E-15 | 1.31 ± 0.12 | 8.44872E-10 |
| 11 mM [U-^13^C_6_]glucose | 13.34 ± 0.54 | 9.97 ± 1.68 | 16.88 ± 2.48 | 3.39827E-10 | 15.43 ± 0.57 | 4.59994E-22 |
| 50 mM [U-^13^C_3_]glycerol | 20.42 ± 4.34 | 16.38 ± 8.84 | 28.97 ± 3.19 | 6.71206E-14 | 18.61 ± 2.66 | 0.15522829 |

***** Mean values were calculated from matrix calculations (resulting in 36 data points) for possible **His/Glu** ratios with six data sets for His and Ala, respectively (2 biological and 2 x 3 technical replicates). The standard deviation was calculated from the resulting 36 **His/Glu** ratios. Statistical analysis was performed using two-tailed unpaired Student´s t-test for the analysis of differences between the wild-type and the *csrA* mutant at E and PE growth phase. Statistical significance is represented as p-value (*p < 0.05, ** p < 0.01 and ***p < 0.001).

**Table S9**: **^13^C-Excess (mol%) of protein-derived amino acids. diaminopimelic acid (DAP). poly-hydroxybutyrate (PHB). lactic acid (LACT) and stearic acid (STE) from experiments with *L. pneumophila* wild-type and its *csrA* mutant grown in CE MDM supplemented with 0.8 mM [1.2.3.4-^13^C_4_]palmitic acid*.**

| 0.8 mM [1.2.3.4-^13^C_4_]palmitic acid | **WT E** | **WT PE** | ***ΔcsrA* E** | **Statistical significance of ΔCsrA E compared to WT E (p-value)**** | ***ΔcsrA* PE** | **Statistical significance of *ΔcsrA* PE compared to WT PE (p-value)**** |
| --- | --- | --- | --- | --- | --- | --- |
| **Ala** | 0.14% ± 0.07% | 0.12% ± 0.05% | 0.19% ± 0.05% |  | 0.14% ± 0.03% |  |
| **Asp** | 0.27% ± 0.05% | 0.17% ± 0.06% | 0.44% ± 0.09% | 0.004036 | 0.23% ± 0.04% | 0.073505 |
| **Glu** | 0.50% ± 0.03% | 0.52% ± 0.06% | 0.78% ± 0.08% | 0.000179 | 0.54% ± 0.07% | 0.620580 |
| **Gly** | 0.06% ± 0.06% | 0.10% ± 0.06% | 0.07% ± 0.04% |  | 0.07% ± 0.07% |  |
| **His** | 0.13% ± 0.03% | 0.15% ± 0.09% | 0.16% ± 0.09% |  | 0.24% ± 0.14% |  |
| **Ile** | 0.12% ± 0.04% | 0.07% ± 0.03% | 0.14% ± 0.05% |  | 0.07% ± 0.02% |  |
| **Leu** | 0.07% ± 0.03% | 0.01% ± 0.01% | 0.12% ± 0.07% |  | 0.02% ± 0.01% |  |
| **Lys** | 0.22% ± 0.07% | 0.18% ± 0.09% | 0.22% ± 0.07% |  | 0.23% ± 0.05% |  |
| **Phe** | 0.18% ± 0.02% | 0.13% ± 0.04% | 0.19% ± 0.04% |  | 0.22% ± 0.02% |  |
| **Pro** | 0.00% ± 0.00% | 0.03% ± 0.04% | 0.00% ± 0.00% |  | 0.06% ± 0.05% |  |
| **Ser** | 0.17% ± 0.04% | 0.14% ± 0.05% | 0.17% ± 0.26% |  | 0.20% ± 0.04% |  |
| **Tyr** | 0.14% ± 0.06% | 0.15% ± 0.07% | 0.18% ± 0.05% |  | 0.15% ± 0.09% |  |
| **Val** | 0.04% ± 0.03% | 0.06% ± 0.03% | 0.09% ± 0.08% |  | 0.08% ± 0.06% |  |
| **DAP** | 0.18% ± 0.06% | 0.11% ± 0.10% | 0.56% ± 0.23% | 0.008019 | 0.31% ± 0.35% | 0.246180 |
| **PHB** | 2.79% ± 1.52% | 3.36% ± 0.91% | 4.93% ± 0.35% | 0.014934 | 6.32% ± 0.76% | 0.000113 |
| **LACT** | 0.16% ± 0.06% | 0.28% ± 0.29% | 0.16% ± 0.19% |  | 0.30% ± 0.18% |  |
| **STE** | 0.19% ± 0.02% | 0.35% ± 0.23% | 0.41% ± 0.12% | 0.006362 | 0.45% ± 0.05% | 0.33576 |

* Cells were harvested at E phase (OD_600_ = 0.35) and PE phase (17d). ** Mean and standard deviation from two independent experiments. Statistical analysis was performed using two-tailed unpaired Student´s t-test for the analysis of differences between the wild-type and the *csrA* mutant at E and PE growth phase. Statistical significance is represented as p-value (*p < 0.05, ** p < 0.01 and ***p < 0.001).

**Table S10:** **Relative fractions of isotopologues (mol%) of PHB and glutamic acid from *L. pneumophila* WT and its *csrA* mutant grown in CE MDM supplemented with 0.8 mM [1.2.3.4-^13^C_4_]palmitic acid.**

|  | | | | |
| --- | --- | --- | --- | --- |
|  | **WT E** | **WT PE** | ***ΔcsrA* E** | ***ΔcsrA* PE** |
| **M+1*** | 1.93% ± 2.01% | 1.21% ± 1.12% | 1.67% ± 2.03% | 1.02% ± 0.59% |
| **M+2** | 3.32% ± 2.09% | 4.60% ± 1.11% | 7.65% ± 1.14% | 10.49% ± 1.26% |
| **M+3** | 0.17% ± 0.37% | 0.51% ± 0.66% | 0.09% ± 0.18% | 0.15% ± 0.22% |
| **M+4** | 0.52% ± 0.51% | 0.38% ± 0.38% | 0.62% ± 0.57% | 0.71% ± 0.33% |
| **Glutamic acid** | | | | |
|  | **WT E** | **WT PE** | ***ΔcsrA* E** | ***ΔcsrA* PE** |
| **M+1** | 0.33% ± 0.18% | 0.30% ± 0.17% | 0.83% ± 0.30% | 0.41% ± 0.21% |
| **M+2** | 0.89% ± 0.12% | 1.05% ± 0.20% | 1.22% ± 0.22% | 0.99% ± 0.19% |
| **M+3** | 0.11% ± 0.03% | 0.01% ± 0.03% | 0.14% ± 0.02% | 0.03% ± 0.03% |
| **M+4** | 0.00% ± 0.00% | 0.01% ± 0.02% | 0.02% ± 0.02% | 0.01% ± 0.02% |
| **M+6** | 0.01% ± 0.01% | 0.02% ± 0.01% | 0.03% ± 0.01% | 0.03% ± 0.01% |

* M+X represents the mass of the unlabeled metabolite plus X labeled ^13^C-atoms. Shown are mean and standard deviation from two independent experiments.

**Table S11: Composition of CE MDM**

| **Compound** | **CE MDM [mg/l]** |
| --- | --- |
| ACES (N-(2-acet-amido)-2-amino-ethanesulfonamid acid) | 10000 |
| Arginine | 350 |
| Cysteine | 400 |
| Isoleucine | 470 |
| Leucine | 640 |
| Methionine | 200 |
| Threonine | 330 |
| Valine | 480 |
| Serine (6mM) | 650 |
| Proline | 115 |
| Phenylalanine | 350 |
| Glycerol (50mM) | 4605 (3654mL) |
| Glucose (11mM) | 1982 |
| NH_4_Cl | 315 |
| NaCl | 50 |
| CaCl_2_ x 2H_2_O | 25 |
| KH_2_PO_4_ | 1180 |
| MgSO_4_ x 7H_2_O | 70 |
| Fe-pyrophosphate hydrate (Fe_4_O_21_P_6_) | 250 |

**Table S12. Retention time and mass fragments of derivatized metabolites used for isotopologue calculations.**

| Metabolite | RT ^a^ [min] | [M-15]^+^ | [M-57]^+^ | [M-85]^+^ | [M-176]^+^ |
| --- | --- | --- | --- | --- | --- |
| **Ala** | 6.7 |  | m/z 260 |  |  |
| **Asp** | 15.4 |  | m/z 418 |  |  |
| **Glu** | 16.8 |  | m/z 432 |  |  |
| **Gly** | 7.0 |  | m/z 246 |  |  |
| **Ile** | 9.5 |  |  | m/z 274 |  |
| **Leu** | 9.1 |  |  | m/z 274 |  |
| **Lys** | 18.1 |  | m/z 431 |  |  |
| **Phe** | 14.5 |  | m/z 336 |  |  |
| **Pro** | 10.1 |  | m/z 286 |  |  |
| **Ser** | 13.2 |  | m/z 390 |  |  |
| **Tyr** | 21.0 |  | m/z 466 |  |  |
| **Val** | 8.5 |  | m/z 288 |  |  |
| **DAP** | 6.3 |  | m/z 589 |  |  |
| **PHB** | 9.1 | m/z 233 |  |  |  |
| **His** | 20.4 |  | m/z 440 |  |  |
| **Man** | 8.7 | m/z 287 |  |  |  |
| **GlcN** | 32.6 | m/z 452 |  |  |  |
| **Mur** | 36.7 |  |  |  | m/z 434 |

^a^ RT, retention time

**Table S13. Statistical analyses of the oxygen consumption rate in *Legionella* depicted in Figure 1**

The values represent the average of n=6 biological replicates performed for each experiment. *p-value ≤ 0.05; **p-value ≤ 0.01; ***p-value ≤ 0.001; ns = non-significant (unpaired t-test). Similarly to Figure 1, the red line indicates the moment of the automatic addition of the substances.


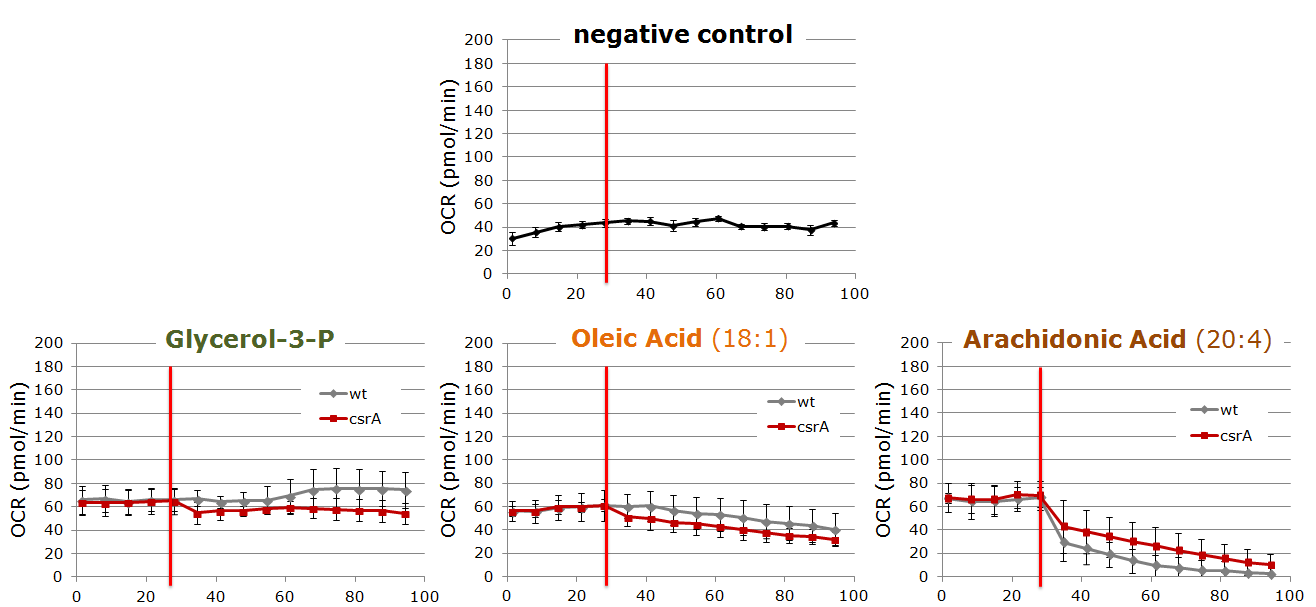


**FIGURE S1. Oxygen consumption experiments.** Changes in bacterial respiration (oxygen consumption rate) of *Legionella pneumophila* wt (grey) and *csrA* mutant (red) were measured as a function of various carbon sources. The final concentration of the different substances added was as follows: glycerol-3-P 0.2 g/L, oleic acid and arachidonic acid 0.1 g/L. As negative control, PBS buffer was added to the wt strain instead of an external carbon source.

**FIGURE S2. ^13^C Isotopologue distributions from experiments with ^13^C-serine as precursor.** Shown are relative isotopologue distributions (mol%) in all significantly enriched metabolites (^13^C-excess > 0.5 mol%) of *L. pneumophila* wild-type **(A)** and the *csrA*-strain **(B)** grown with 6 mM [U-^13^C_3_]serine as tracer. Bacteria where grown in CE MDM and harvested at E phase and PE phase. Isotopologue distributions where calculated by isotopologue profiling. Shown are relative fractions (%) of isotopologues (M+1 to M+7) calculated from two independent experiments. For numerical values, see **Table S2**.

**FIGURE S3. ^13^C Isotopologue distributions from experiments with ^13^C-glucose as precursor.** Shown are relative isotopologue distributions (%) in all significantly enriched metabolites (^13^C-excess > 0.5 mol%) of *L. pneumophila* wild-type **(A)** and the *csrA*-strain **(B)** grown with 11 mM [U-^13^C_6_]glucose as tracer. Bacteria where grown in CE MDM and harvested at E phase and PE phase. Isotopologue distributions where calculated by isotopologue profiling. Shown are relative fractions (%) of isotopologues (M+1 to M+7) calculated from two independent experiments. For numerical values, see **Table S4.**

**FIGURE S4. ^13^C Isotopologue distributions from experiments with ^13^C-glycerol as precursor.** Shown are relative isotopologue distributions (%) in all significantly enriched metabolites (^13^C-excess > 0.5 mol%) of *L. pneumophila* wild-type **(A)** and the *csrA*-strain **(B)** grown with 50 mM [U-^13^C_3_]glycerol as tracer. Bacteria where grown in CE MDM and harvested at E phase and PE phase. Isotopologue distributions where calculated by isotopologue profiling. Shown are relative fractions (%) of isotopologues (M+1 to M+7) calculated from two independent experiments. For numerical values, see **Table S6.**


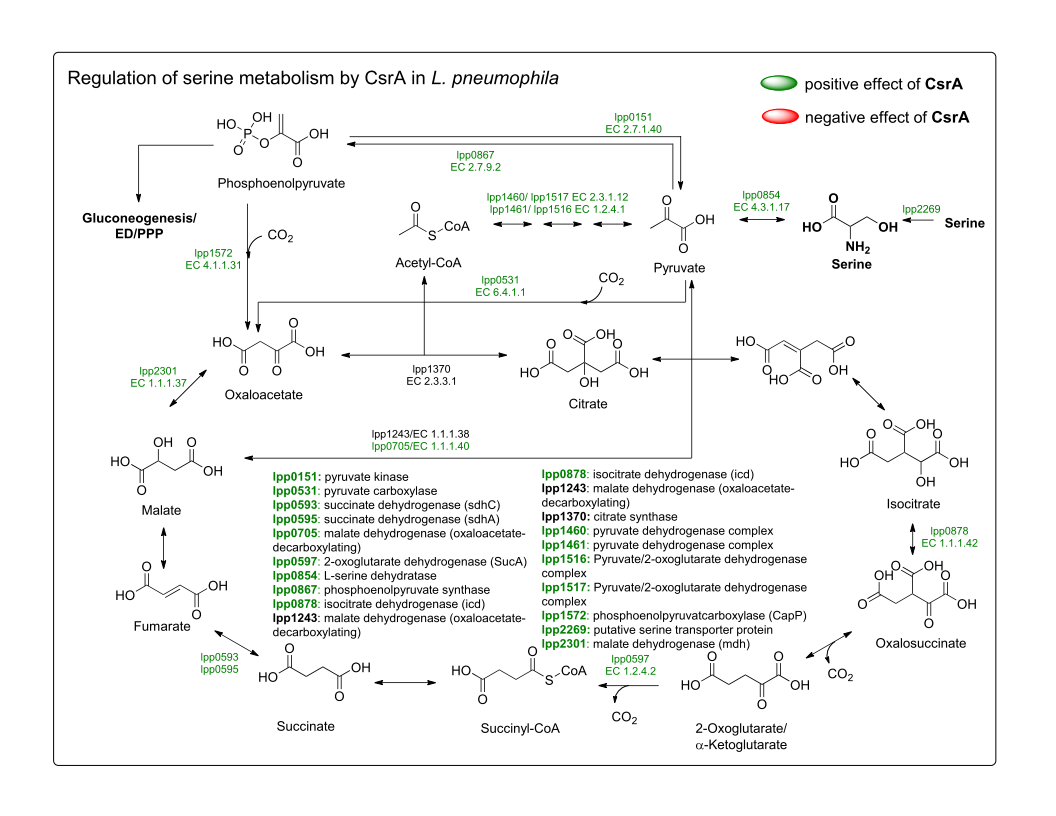


**FIGURE S5. Regulation of serine metabolism by CsrA in *L. pneumophila*.** Previous extensive transcriptome and proteome experiments in combination with RNA-Co-immunoprecipitation experiments followed by deep sequencing of a *L. pneumophila* wt and a *csrA* mutant strain revealed 516 RNAs, which are affected by this central regulator [1]. This figure illustrates the regulatory effects of CsrA on enzymes in the metabolism of serine in the TCA cycle. Thereby, positive effects of CsrA are indicated in green whereas negative effects are indicated in red. This figure shows, that CsrA has a positive effect on enzyme of the TCA cycle as well as serine incorporation during E phase of *L. pneumophila*. The illustrated positive and negative effects of CsrA are based on previously reported transcriptome and proteome data [1].

**FIGURE S6. Regulation of glucose and glycerol metabolism by CsrA in *L. pneumophila*.** Previous extensive transcriptome and proteome experiments in combination with RNA-Co-immunoprecipitation experiments followed by deep sequencing of a *L. pneumophila* wild-type and a *csrA* mutant revealed 516 RNAs, which are affected by this central regulator ([1]). This figure illustrates the regulatory effects of CsrA on enzymes in the metabolism of glucose and glycerol in the ED pathway, glycolysis, gluconeogenesis and the pentose phosphate pathway. Thereby, positive effects of CsrA are indicated in green whereas negative effects are indicated in red. This figure illustrates that CsrA has a positive effect on enzymes of the ED pathway, glycolytic and gluconeogenetic reactions during E growth phase. Negative effects of CsrA have been observed for glucose uptake and glycerol metabolism. The illustrated positive and negative effects of CsrA are based on previously reported transcriptome and proteome data [1].

**FIGURE S7. Regulation of PHB metabolism by CsrA in *L. pneumophila*.** Previous extensive transcriptome and proteome experiments in combination with RNA-Co-immunoprecipitation experiments followed by deep sequencing of a *L. pneumophila* wild-type and a *csrA* mutant revealed 516 RNAs, which are affected by this central regulator [1]. This figure illustrates the regulatory effects of CsrA on enzymes involved in the biosynthesis of PHB. Thereby, positive effects of CsrA are indicated in green whereas negative effects are indicated in red. This figure illustrates that CsrA has a negative effect on enzymes of PHB biosynthesis during E growth phase. The illustrated positive and negative effects of CsrA are based on previously reported transcriptome and proteome data [1].

**References**

1 Sahr, T., Rusniok, C., Impens, F., Oliva, G., Sismeiro, O., Coppee, J. Y., Buchrieser, C. 2017 The *Legionella pneumophila* genome evolved to accommodate multiple regulatory mechanisms controlled by the CsrA-system. *PLoS Genet*. **13**, e1006629. (10.1371/journal.pgen.1006629)
